# Fecal Carriage of Multidrug‐Resistant *Staphylococcus aureus* in Hypertensive Patients at the Douala Laquintinie Hospital: Prevalence and Resistance Patterns

**DOI:** 10.1155/bmri/8076503

**Published:** 2025-11-26

**Authors:** Ornella Djiolieu Tsobeng, Armelle T. Mbaveng, Michael F. Kengne, Ballue S. T. Dadjo, Victor Kuete

**Affiliations:** ^1^ Department of Biochemistry, Faculty of Science, Université de Dschang, Dschang, Cameroon, univ-dschang.org

**Keywords:** antibiotic resistance, fecal, hypertension, *S. aureus*

## Abstract

Patients with hypertension may be more susceptible to acquiring *Staphylococcus aureus* (*S. aureus*) infections, according to some studies. Hypertension and certain antihypertensive drugs predispose to multidrug‐resistant bacteria. The present study, carried out at the Laquintinie Hospital in Douala, is aimed at determining the antibiotic‐resistant profile of fecal carriage *S. aureus* in hypertensive patients and their association with hypertension. This was a cross‐sectional study that was carried out from June 2022 to June 2023. Five hundred and eighteen (518) stool samples were collected, from which the isolation of *S. aureus* was made using mannitol salt agar. Mannitol fermentation, catalase, and coagulase tests were used for species identification. The Kirby–Bauer disc diffusion method was used for the antibiotic susceptibility assay. Our study revealed that the frequency of fecal carriage of *S. aureus* was significantly higher in hypertensive participants (65.15%, *n* = 43) compared to nonhypertensive participants (34.85%, *n* = 23). The frequency of fecal carriage of methicillin‐resistant *S. aureus* (MRSA) was significantly higher in participants with hypertension compared to nonhypertensive participants (88.37% vs. 47.83%, *p* ~ 0.001). The antibiotic susceptibility test revealed that the resistance of *S. aureus* to fusidic acid, cotrimoxazole, and oxacillin was significantly higher in hypertensive than in nonhypertensive patients. There was a significant association between hypertension and *S. aureus* resistance to oxacillin (OR = 8.29, *p* ~ 0.001) and trimethoprim–sulfamethoxazole (OR = 6.07, *p* = 0.001). In addition, *S. aureus* isolates showed high resistance rates in treated hypertensive participants compared to untreated hypertensive participants. This study reveals that *S. aureus* exhibits high resistance to many of the clinically used antimicrobials. The need for appropriate antibiotic use to halt, or at least limit, the spread of resistance is suggested in the care of hypertensive patients with enteric infection caused by *S. aureus*.

## 1. Introduction

*Staphylococcus aureus* is a major bacterial pathogen that poses a significant threat to public health due to its ability to cause severe infections and its increasing resistance to antibiotics [1]. This bacterium is a major human pathogen responsible for a wide range of infections, including gastrointestinal tract infections, among the population with weak immune systems [2–5]. Many studies show that individuals with hypertension have a risk of intestinal microbiota dysbiosis, characterized by reduced bacterial biodiversity [6–8]. This dysbiosis can lead to increased intestinal permeability, allowing pathogenic bacteria to enter the bloodstream and cause infections [9]. Hypertension can also lead to chronic inflammation, which can weaken the immune system and increase susceptibility to *S. aureus* infections [10, 11]. As a result, individuals with hypertension may be more vulnerable to *S. aureus* infections, including those caused by resistant bacteria such as methicillin‐resistant *S. aureus* (MRSA) [12]. Furthermore, some studies suggest that certain antihypertensive medications may increase the risk of MRSA [13, 14]. MRSA is a specific strain of the *S. aureus* bacterium that has developed antimicrobial resistance to penicillin, including methicillin and other narrow‐spectrum beta‐lactamase‐resistant penicillin antimicrobials [15]. MRSA has since emerged as a serious concern in human medicine. Although these organisms cause the same types of infections as other *S. aureus* strains, hospital‐associated strains have become resistant to the most common antimicrobials, making treatment a great challenge [16]. Antimicrobial resistance is a long‐standing problem with the magnitude and speed at which it spreads, becoming globally one of the most serious current public health problems [17, 18]. The determination of the prevalence of *S. aureus* antibiotic resistance in the hypertensive population has not been studied in Cameroon. Most fecal carriage of *S. aureus* studies appear to have focused on the general population [19] and a few risk groups, such as HIV‐infected persons [2], sickle cell disease patients [4], pregnant women [5], and cancer patients [20]. In Cameroon, the prevalence of *S. aureus* among hypertensive patients and the resistance profile of *S. aureus* epidemiology are less well known. Given the high burden of hypertension and its potential impact on susceptibility to infections, this study is crucial to determine the antibiotic resistance profile of fecal carriage *S. aureus* in hypertensive patients and their association with hypertension. This study addresses a critical knowledge gap on the prevalence and resistance profiles of *S. aureus* in hypertensive patients. Hypertension is a common medical condition that can increase susceptibility to infections, and hypertensive patients may be particularly vulnerable to complications of *S. aureus* infections [9, 11–13]. By determining the links between hypertension and *S. aureus* infections, this study can contribute to improving patient care and informing public health strategies to combat antimicrobial resistance. The aim of this study, therefore, was to determine the antibiotic‐resistant profile of fecal carriage *S. aureus* in hypertensive patients and their association with hypertension.

## 2. Materials and Methods

### 2.1. Study Design, Population Studied, and Selection Criteria

This was a cross‐sectional study carried out from June 2022 to June 2023 at the Laquintinie Hospital in Douala, located in the Littoral region of Cameroon. The study recruited both hypertensive and nonhypertensive patients based on clearly defined inclusion criteria. Participants were identified within the cardiology and gastroenterology units of Laquintinie Hospital in Douala. Only individuals diagnosed with enteric infections linked to *Staphylococcus aureus* and who provided informed consent were enrolled in the research. Nonhypertensive individuals were identified and selected based on the following criteria: systolic blood pressure (SBP) less than 140 mmHg and diastolic blood pressure (DBP) less than 90 mmHg, confirmed by ambulatory blood pressure monitoring. Additionally, we excluded individuals who had received antibiotic treatment within the 2 weeks preceding recruitment, those who tested positive for HIV, pregnant women, patients with confirmed Hepatitis B or C serology, and those infected with bacterial genera other than *S. aureus*.

Sample size calculation is as follows:

The sample size (*N*) was calculated using the LORENZ formula:

N=z2×p 1−pm2.

Details include the following:

*N*: sample size required for this study.

*z*: 95% confidence level (standard value of 1.96).

*p*: estimated prevalence of hypertension in Cameroon, 30.9% [21];

*m*: margin of error at 5% (standard value 0.05).

Based on the prevalence of hypertension of 30.9% in the Cameroonian population [21], the theoretically recommended sample size was 328 participants according to the Lorenz formula. However, we were able to recruit 518 participants, thus exceeding the recommended threshold, which could improve the statistical power and precision of the estimates. After stool culture, 66 patients were confirmed to have enteric infections caused by *S. aureus* and were included in this study, while 452 patients infected with other bacterial genera were excluded.

### 2.2. Ethical Approval

Ethical approval for this study was granted by the Institutional Ethics Committee for Research on Human Health at the University of Douala (Littoral, Cameroon) (CEI‐UDo), under reference number 3130CEI‐UDo/06/2022/T. In addition, administrative permission was obtained from the director of Laquintinie Hospital. All adult participants received detailed information sheets explaining the study′s objectives and procedures, and each provided written informed consent prior to enrollment.

### 2.3. Blood Pressure Measurement

Blood pressure was measured in both participants, previously diagnosed with hypertension by a cardiologist, and those without a known history of hypertension. To ensure reliable readings, each participant was asked to rest in a seated position for at least 5 min prior to the measurement. A full‐screen electronic sphygmomanometer was used, with the cuff placed on the left arm. To reduce variability, three measurements were taken at 10‐min intervals, and the average value was recorded. Hypertension was defined according to the European Society of Hypertension criteria as a SBP ≥ 140 mmHg and/or a DBP ≥ 90 mmHg. Based on the recorded values, participants were categorized into the following hypertension grades: Grade 1 (mild) hypertension: SBP = 140–159 mmHg and/or DBP = 90–99 mmHg; Grade 2 (moderate) hypertension: SBP = 160–179 mmHg and/or DBP = 100–109 mmHg; and Grade 3 (severe) hypertension: SBP ≥ 180 mmHg and/or DBP ≥ 110 mmHg [22, 23].

### 2.4. Sociodemographic Data Collection and Stool Sample Collection

Sociodemographic data such as age, gender, marital status, level of education, and data on hypertension (hypertension status, types of antihypertensive medication used, and family history of hypertension) were collected using a research questionnaire.

A total of 518 fresh stool samples were obtained from patients for whom a physician had prescribed a coproculture. Prior to sample collection, it was confirmed that participants were not under antibiotic treatment and had followed specific precollection guidelines: Wash hands thoroughly, urinate beforehand, and ensure that the stool sample was not contaminated with urine. Each specimen was prelabelled and placed in sterile, leak‐proof plastic containers (Viamed, Miami Lakes, Florida) and then transported to the microbiology laboratory for culture within 2 h of collection [24].

### 2.5. Isolation and Identification of *Staphylococcus aureus* From Stool

Clinical specimens were inoculated onto mannitol salt agar (MSA) plates and incubated at 37°C for 24 h. Colonies obtained from the primary culture were then purified by subculturing onto fresh MSA plates, followed by another 24‐h incubation at 37°C. Smears were prepared from isolated colonies on clean, grease‐free glass slides and stained using Gram′s method. Microscopic examination revealed gram‐positive cocci arranged in irregular clusters resembling grape bunches. The tube coagulase test was conducted by mixing bacterial colonies with 250 *μ*L of rabbit plasma containing EDTA in small test tubes, which were incubated for 24 h. Catalase activity was assessed by applying a drop of hydrogen peroxide onto a slide and introducing an isolated colony collected with a sterile plastic loop; the presence of oxygen bubbles indicated catalase positivity. These biochemical assays confirmed *Staphylococcus aureus* identification, characterized by coagulase and catalase positivity, and the formation of yellowish colonies on MSA [20].

### 2.6. Antibiotic Susceptibility Test

The susceptibility of *S. aureus* isolates was assessed using the Kirby–Bauer disk diffusion technique, following standard quality control procedures [25]. A panel of commonly used antibiotics was tested, including cefoxitin, oxacillin, ciprofloxacin, gentamicin, tobramycin, tetracycline, minocycline, doxycycline, clindamycin, erythromycin, azithromycin, fusidic acid, nitrofurantoin, and cotrimoxazole (Singapore Biosciences PTE Ltd., Singapore). Quality control of antibiotics was ensured using reference strains such as *S. aureus* ATCC 29213 (methicillin‐susceptible) and *S. aureus* ATCC 33591(methicillin‐resistant). These reference strains were used to validate the antibiotic susceptibility tests by being included in each series of tests performed with the bacterial isolates. For each class of antibiotics tested, the *S. aureus* ATCC 29213 and ATCC 33591 strains were tested in parallel with the bacterial isolates to ensure that the results obtained were accurate and reliable according to CLSI criteria. The inhibition zones measured for these reference strains were compared to expected values to validate the test results [26]. Bacterial isolates were suspended in physiological saline to achieve turbidity comparable to the 0.5 McFarland standard and then inoculated onto Mueller–Hinton agar (MHA) plates. Antibiotic disks were placed on the agar surface, and plates were incubated at 37°C for 24 h. The resulting inhibition zones were measured and interpreted following the Clinical and Laboratory Standards Institute (CLSI) guidelines [27]. Methicillin resistance was assessed using a 30 *μ*g cefoxitin disk, with an inhibition zone diameter of 25 mm or less indicating resistance. Isolates resistant to three or more antibiotic classes were classified as multidrug‐resistant (MDR) bacteria [28, 29].

### 2.7. Statistical Analysis

This study examined antibiotic resistance patterns in *Staphylococcus aureus* and their potential association with hypertension. Data were initially entered into Microsoft Excel and subsequently imported into Epi Info Version 7.2.4 (CDC, Atlanta, United States) for statistical analysis. The prevalence of *S. aureus* infection was determined by calculating the proportion of positive isolates among the 518 stool samples analyzed, with a 95% confidence interval (CI). Patient characteristics, the distribution of *S. aureus* according to antihypertensive drug use, and antimicrobial susceptibility profiles were described using frequencies and percentages. The chi‐square (*χ* ^2^) test was used to compare resistance and MDR rates between hypertensive and nonhypertensive participants. To assess the association between antibiotic resistance and hypertension, logistic regression analysis was performed. The strength of these associations was estimated using odds ratios (ORs) with corresponding 95% CIs. A significance level of 5% was adopted, and results with *p* values less than 0.05 were considered statistically significant.

## 3. Results

### 3.1. Sociodemographic Characteristics of the Study Population

Of the 518 patients included in the study, 211 (40.3%) were male, and 307 (59.7%) were female. Hypertension was more frequent among women, with 197 cases (60.24%) compared to 130 cases (39.76%) among men, indicating a significant difference in distribution between sexes. Hypertensive patients had a significantly higher mean age than nonhypertensive patients (59.54 ± 12.14 vs. 43.45 ± 15.54; *p* ~ 0.001). Furthermore, widowed participants were more likely to have hypertension (*p* ~ 0.001), whereas single participants were less affected. Additionally, participants with a primary level of education were more likely to suffer from hypertension (*p* ~ 0.001) compared to those with a higher education level. Detailed results are presented in Table 1. The characteristics of the study participants are summarized in Table S1.

**Table 1:** Demographic profile of hypertensive and nonhypertensive patients.

**Parameters**		**HTA+ (** **n** = 327**, 63.13%)**	**HTA− (** **n** = 191**, 36.87%)**	**p** **value**
Sex	Male (*n* = 211)	130 (39.76)	81 (42.41)	0.553
	Female (*n* = 307)	197 (60.24)	110 (57.59)	

Mean age (years); mean ± SD [min–max]	Total	59.54 ± 12.14 [32–88]	43.45 ± 15.54 [20–87]	~0.001
	Male	58.13 ± 12.18 [32–87]	43.30 ± 13.43 [20–81]	~0.001
	Female	60.46 ± 12.05 [33–88]	43.56 ± 16.98 [20–84]	~0.001

Blood pressure [min–max]	SBP; mean ± SD (mmHg)	165.44 ± 25.74 [102–269]	119.30 ± 11.36 [80–152]	~0.001
	DBP; mean ± SD (mmHg)	99.63 ± 17.94 [58–117]	77.57 ± 11.12 [49–110]	~0.001
	Pulsation; mean ± SD (bpm)	87.20 ± 16.40 [42–154]	82.71 ± 13.83 [46–110]	0.001

Matrimonial status	Single (*n* = 87)	36 (11.01)	51 (26.70)	~0.001
	Married (*n* = 322)	205 (62.08)	117 (61.26)	0.724
	Widow (er) (*n* = 109)	86 (26.30)	23 (12.04)	~0.001

Level of education	Illiterate (*n* = 43)	31 (9.48)	12 (6.28)	0.167
	Elementary school (*n* = 121)	93 (28.44)	28 (14.66)	~0.001
	Secondary school (*n* = 303)	181 (55.35)	122 (63.87)	0.069
	Higher education (*n* = 51)	22 (6.73)	29 (15.18)	0.001

Family history of hypertension	Yes (*n* = 289)	219 (66.67)	70 (36.87)	~0.001
	No (*n* = 229)	108 (33.03)	121 (63.35)	

*Note:* Hypertension was characterized by SBP ≥ 140 mmHg and PAS ≥ 80 mmHg. Duration of hypertension varied from 1 to 35 years.

Abbreviations: bpm, beats per minute; DBP, diastolic blood pressure; HTA+, hypertensive patients; HTA−, nonhypertensive patients; max, maximum; min, minimum; *n*, number of participants; SBP, systolic blood pressure.

### 3.2. Distribution of Types of Therapy and Antihypertensive Drugs According to Hypertension Grade

Table 2 presents the distribution of antihypertensive medications and treatment types according to hypertension grade. The hypertensive population was categorized into two groups: treated and untreated hypertensive patients. Among untreated hypertensive patients, Grade 3 hypertension was the most prevalent (42.17%), followed by Grades 1 and 2 (28.92%). In contrast, among treated hypertensive patients, Grade 1 hypertension (38.11%) and Grade 3 hypertension (37.70%) were the most common. Notably, significant differences were observed in the prescription rates of antihypertensive drugs according to hypertensive grade (*p* ~ 0.001). Specifically, monotherapy was predominantly prescribed to patients with Grade 1 hypertension (41.52%), whereas bitherapy (44.44%) and tritherapy (60.00%) were more commonly prescribed to patients with Grade 3 hypertension. Furthermore, the distribution of beta‐blockers, diuretics, calcium channel blockers (CCBs), CCB+diuretics, ACEI+diuretics, and triplexam varied significantly according to hypertension grade (*p* ~ 0.001). The hypertension status and treatment profiles of the study participants are summarized in Table S2.

**Table 2:** Distribution of antihypertensive therapy and drugs according to hypertension grade.

**Characteristics**	**Hypertension**			**p** **value**
	**Grade 1,** **n** **(%)**	**Grade 2,** **n** **(%)**	**Grade 3,** **n** **(%)**	
Treated hypertensive patients	24 (28.92)	24 (28.92)	35 (42.17)	~0.001 ^∗^
Untreated hypertensive patients	93 (38.11)	59 (24.18)	92 (37.70)	
Hypertension therapy				
Monotherapy	71 (41.52)	42 (26.56)	58 (33.92)	~0.001 ^∗^
Bitherapy	20 (31.75)	15 (23.81)	28 (44.44)	
Tritherapy	2 (20.00)	2 (20.00)	6 (60.00)	
Classes of antihypertensive drugs				
Angiotensin II receptor blocker (ARB2)	1 (33.22)	1 (33.22)	1 (33.22)	0.587
ARB2+diuretic	1 (50.00)	0 (0.00)	1 (50.00)	0.519
Beta‐blocker	17 (39.53)	9 (20.93)	17 (39.53)	~0.001 ^∗^
Diuretic	17 (49.59)	9 (23.08)	13 (33.33)	~0.001 ^∗^
Calcium channel blockers (CCBs)	51 (40.16)	30 (22.83)	48 (37.01)	~0.001 ^∗^
CCB+diuretic	8 (30.77)	8 (30.77)	10 (38.46)	~0.001 ^∗^
Angiotensin‐converting enzyme inhibitor (ACEI)	5 (50.00)	2 (20.00)	3 (30.00)	0.061
ACEI+diuretic	14 (35.00)	8 (20.00)	18 (45.00)	~0.001 ^∗^
Triplixam	5 (33.33)	2 (13.33)	8 (53.33)	0.008 ^∗^

*Note:* *n*, frequency. Grade 1: systolic 140–159 mmHg and/or diastolic 90–99 mmHg. Grade 2: systolic 160–179 mmHg or greater and/or diastolic 100–109 mmHg. Grade 3: systolic 180 mmHg or greater and/or diastolic 110 mmHg or greater. The duration of hypertensive medications varied from 1 to 22 years.

^∗^Significant distribution of antihypertensive medications.

### 3.3. Association Between Blood Pressure and Fecal Carriage of *S. aureus* in Hypertensive and Nonhypertensive Patients

Table 3 presents the association between the distribution of *S. aureus* in hypertensive and nonhypertensive patients and blood pressure. The results indicate that *S. aureus* infection was significantly more prevalent in hypertensive patients than in nonhypertensive patients with SBP of 140–159 mmHg (*p* ~ 0.001), 160–179 mmHg (*p* ~ 0.001), and ≥ 180 mmHg (*p* = 0.018). In contrast, *S. aureus* was significantly more frequently isolated in nonhypertensive patients with SBP less than 140 mmHg (*p* ~ 0.001). Furthermore, nonhypertensive patients with a DBP less than 90 mmHg had a 13.80‐fold increased risk of *S. aureus* infection.

**Table 3:** Association between fecal carriage of *S. aureus* and blood pressure.

**Blood pressure**	**HTN+,** **n** **(%)**	**HTN−,** **n** **(%)**	**X** ^2^	**p** **value**	**OR (95% CI)**
Systolic blood pressure					
< 140 mmHg	6 (13.95)	23 (100)	45.04	~0.001 ^∗^	ND
140–159 mmHg	16 (37.21)	0 (0.00)	11.29	~0.001 ^∗^	ND
160–179 mmHg	12 (27.91)	0 (0.00)	7.84	~0.001 ^∗^	ND
≥ 180 mmHg	9 (20.93)	0 (0.00)	5.57	0.018 ^∗^	ND

Diastolic blood pressure					
< 90 mmHg	14 (32.56)	20 (86.96)	17.75	~0.001 ^∗^	13.80 (3.50–54.39)
90–99 mmHg	8 (18.60)	2 (8.70)	1.14	0.284	0.41 (0.08–2.15)
100–109 mmHg	12 (27.91)	0 (0.00)	7.84	0.005 ^∗^	ND
≥ 110 mmHg	9 (20.93)	1 (4.35)	3.20	0.073	0.17 (0.02–1.45)

*Note:* The *p* value is given at a 95% confidence interval and is significant at < 0.05. Hypertension was characterized by SBP ≥ 140 mmHg and DBP ≥ 80 mmHg.

Abbreviations: CI, confidence interval; HTN+, patients with hypertension; HTN−, patients without hypertension; *n*, number of *S. aureus* isolates; ND, not defined; OR, odds ratio.

^∗^Significant association between blood pressure and *S. aureus* infection.

### 3.4. Prevalence and Distribution of *S. aureus* Among Patients According to Sex, Age, Antihypertensive Drug, and Grade

**Figure 1:**
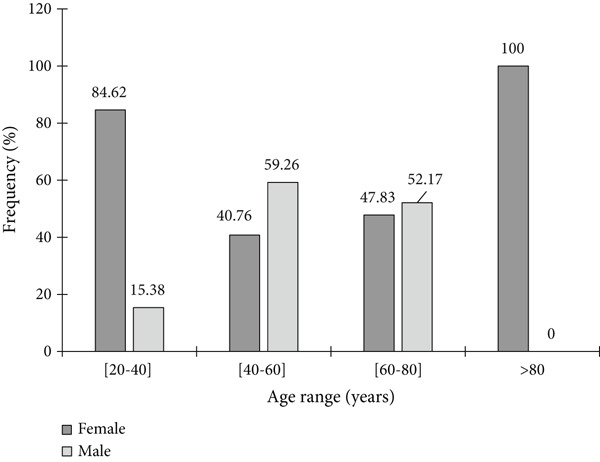
Frequency of *S. aureus* isolates among participants according to gender and age.

Figure 1 presents the distribution of *S. aureus* isolates according to participants′ gender and age. Overall, *S. aureus* was more commonly isolated from female participants (54.55%, *n* = 36) than from males (45.45%, *n* = 30). Age‐specific analysis revealed the highest isolation rates among women aged 20–39 years (84.62%) and those over 79 years (100%). Conversely, in the 40–59 age group, *S. aureus* was more frequently detected in men (59.24%) than in women (40.76%). The overall prevalence of *S. aureus* infection in the study population was 12.74% (*n* = 66/518). Furthermore, *S. aureus* was more frequently isolated in hypertensive patients (65.15%, *n* = 43) compared to nonhypertensive patients (34.84%, *n* = 23). Analysis of antihypertensive drug classes revealed that *S. aureus* was most frequently isolated in patients taking CCB (34.85%, *n* = 23), followed by diuretics (19.15%, *n* = 13) (Table 4).

**Table 4:** Distribution of *Staphylococcus aureus* according to antihypertensive drugs, hypertensive grade, and status.

**Status**	**Parameters**	***Staphylococcus aureus* (** **n** = 66**)**	
		**N**	**Percentage (%)**
Hypertension status	HTA+	43	65.15
	HTA−	23	34.85

Hypertension grade	Grade 1	17	25.76
	Grade 2	11	16.67
	Grade 3	15	22.73

Hypertension therapy	Monotherapy	29	43.94
	Bitherapy	9	13.64
	Tritherapy	1	1.52

Hypertensive class	ACEI	2	3.03
	CCB	23	34.85
	Beta‐blocker	6	9.09
	Diuretic	13	19.15
	ARB2	1	1.52
	Calcium antagonist	2	3.03
	ACEI+CCB	1	1.52
	ACEI+diuretic	6	9.09
	ARB2+diuretic	2	3.03

*Note:* Hypertension was characterized by SBP ≥ 140 mmHg and DBP ≥ 80 mmHg. Grade 1: systolic 140–159 mmHg and/or diastolic 90–99 mmHg. Grade 2: systolic 160–179 mmHg or greater and/or diastolic 100–109 mmHg. Grade 3: systolic 180 mmHg or greater and/or diastolic 110 mmHg or greater.

Abbreviations: ACEI, angiotensin‐converting enzyme inhibitor; ARB2, Angiotensin II receptor blocker; CCB, calcium channel blocker; *N*, number of *Staphylococcus aureus*.

### 3.5. *S. aureus* Antibiotic Susceptibility Profile Among Hypertensive and Nonhypertensive Patients

The antibiotic susceptibility profiles of *S. aureus* isolates are presented in Table 5. The antimicrobial susceptibility test revealed that *S. aureus* isolates from hypertensive patients exhibited significantly higher resistance rates to fusidic acid (*p* = 0.023), cotrimoxazole (*p* ~ 0.001), and oxacillin (*p* ~ 0.001) compared to nonhypertensive patients. Furthermore, *S. aureus* isolates from hypertensive patients showed higher resistance rates to minocycline (81.40% vs. 65.22%) and cefoxitin (67.44% vs. 47.83%) compared to nonhypertensive patients. In nonhypertensive patients, the highest resistance rate was observed for azithromycin (65.22%), while gentamicin exhibited the highest overall sensitivity (86.96%), followed by nitrofurantoin (73.91%) and tobramycin (60.87%). High frequencies of resistance to erythromycin, tetracycline, clindamycin, and doxycycline were observed in both hypertensive (83.72%, 79.07%, 83.72%, and 83.72%, respectively) and nonhypertensive patients (82.61%, 73.91%, 82.61%, and 73%, respectively). The distribution of *S. aureus* infection and the corresponding antibiogram results are detailed in Table S3.

**Table 5:** Susceptibility profile of *Staphylococcus aureus* according to hypertension status.

**Antibiotics**		***Staphylococcus aureus*, n = 66 (%)**		**X** ^2^	**p** **value (between HTN+ and HTN−)**
		**HTN+,** **n** **(%)**	**HTN−,** **n** **(%)**		
Gentamicin	Susceptible	30 (69.77)	20 (86.96)		
	Intermediate	0 (0.00)	0 (0.00)	2.41	0.120
	Resistant	13 (30.23)	3 (13.04)		

Tobramycin	Susceptible	23 (53.49)	14 (60.87)		
	Intermediate	0 (0.00)	0 (0.00)	0.33	0.564
	Resistant	20 (46.51)	9 (39.13)		

Minocycline	Susceptible	7 (16.28)	6 (26.09)		
	Intermediate	1 (2.33)	2 (8.70)	2.58	0.274
	Resistant	35 (81.40)	15 (65.22)		

Tetracycline	Susceptible	6 (13.95)	4 (17.39)		
	Intermediate	3 (6.98)	0 (0.00)	0.22	0.903
	Resistant	34 (79.07)	17 (73.91)		

Clindamycin	Susceptible	7 (16.28)	4 (17.39)		
	Intermediate	0 (0.00)	0 (0.00)	0.01	0.908
	Resistant	36 (83.72)	19 (82.61)		

Erythromycin	Susceptible	5 (11.63)	4 (17.39)		
	Intermediate	2 (4.65)	0 (0.00)	1.43	0.487
	Resistant	36 (83.72)	19 (82.61)		

Fusidic acid	Susceptible	9 (20.93)	11 (47.83)		
	Intermediate	0 (0.00)	0 (0.00)	4.16	**0.023** ^∗^
	Resistant	34 (79.07)	12 (52.17)		

Cefoxitin	Susceptible	14 (32.56)	12 (52.17)		
	Intermediate	0 (0.00)	0 (0.00)	2.41	0.120
	Resistant	29 (67.44)	11 (47.83)		

Nitrofurantoin	Susceptible	19 (44.19)	17 (73.91)		
	Intermediate	0 (0.00)	0 (0.00)	2.08	0.148
	Resistant	24 (55.81)	6 (26.09)		

Cotrimoxazole	Susceptible	15 (34.88)	3 (13.04)		
	Intermediate	0 (0.00)	15 (65.22)	32.27	~0.001 ^∗^
	Resistant	27 (62.79)	5 (21.74)		

Ciprofloxacin	Susceptible	3 (6.98)	15 (62.22)		
	Intermediate	19 (44.19)	3 (13.04)	25.79	~0.001 ^∗^
	Resistant	21 (48.84)	5 (21.74)		

Oxacillin	Susceptible	5 (11.63)	12 (52.17)		
	Intermediate	0 (0.00)	0 (0.00)	12.88	~0.001 ^∗^
	Resistant	38 (88.37)	11 (47.83)		

Doxycycline	Susceptible	7 (16.28)	6 (26.09)		
	Intermediate	0 (0.00)	0 (0.00)	0.91	0.339
	Resistant	36 (83.72)	17 (73.91)		

Azithromycin	Susceptible	13 (30.23)	6 (26.09)		
	Intermediate	7 (16.28)	2 (8.70)	1.07	0.582
	Resistant	23 (53.49)	15 (65.22)		

*Note:* Hypertension was characterized by SBP ≥ 140 mmHg and DBP ≥ 80 mmHg.

Abbreviations: *n*, number of *Staphylococcus aureus* isolates; HTN+, hypertensive patients; HTN−, nonhypertensive patients.

^∗^Significant antibiotic resistance.

### 3.6. Prevalence of MDR *S. aureus*, MRSA, and Methicillin‐Sensitive *Staphylococcus aureus* (MSSA)

**Figure 2:**
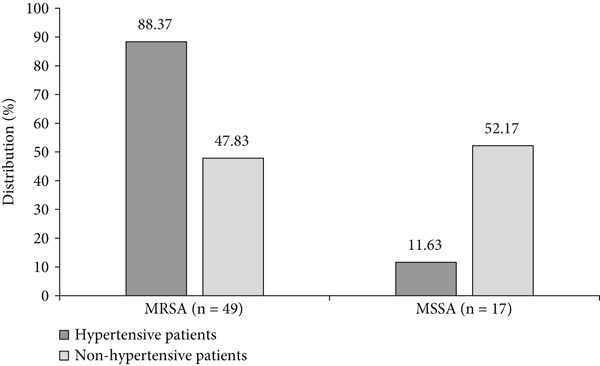
Frequency of methicillin‐resistant *S. aureus* (MRSA) and methicillin‐sensitive *S. aureus* (MSSA) isolated from hypertensive and nonhypertensive patients.  ^∗^ *p* ~ 0.001 and  ^∗∗^ *p* ~ 0.001.

**Figure 3:**
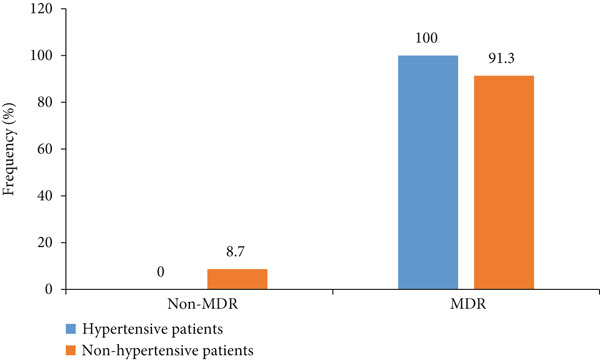
Frequency of multidrug‐resistant (MDR) *Staphylococcus aureus* isolated from hypertensive and nonhypertensive patients.

Of the 66 *S. aureus* isolates, the prevalence of MRSA was 75.75%, higher than that of MSSA (24.25%). The frequency of MRSA was significantly higher (*p* ~ 0.001) in hypertensive patients (88.37%) than in nonhypertensive patients (47.83%). Additionally, the frequency of MSSA was significantly higher (*p* ~ 0.001) in nonhypertension patients (52.17%) compared to hypertensive participants (11.63%) (Figure 2). Figure 3 illustrates the frequency of MDR in *S. aureus* isolates from both hypertensive and nonhypertensive patients. A high proportion of isolates exhibited MDR, with rates of 100% in hypertensive individuals and 91.3% in nonhypertensive individuals. Table 6 summarizes the distribution of MRSA and MSSA based on MDR status and the use of antihypertensive medications. All MRSA isolates were MDR with a significant difference compared to MSSA (100% vs. 87.50%; *p* = 0.011). Furthermore, patients taking CCB were more likely to be infected with MRSA than MSSA (*p* = 0.003).

**Table 6:** Fecal carriage of *Staphylococcus aureus* among participants with regard to MDR and the antihypertensive drugs used in their treatment.

**Status**	**Parameters**	**MRSA (** **N** **(%))**	**MSSA (** **N** **(%))**	**p** **value (between MRSA and MSSA)**
MDR profile	MDR	50 (100)	14 (87.50)	0.011
	Non‐MDR	0 (0.00)	2 (12.50)	

Antihypertensive medications	ACEI (*N* = 2)	2 (100)	0 (0.00)	0.397
	CCB (*N* = 25)	23 (95.65)	2 (4.35)	0.003
	Beta‐blocker (*N* = 6)	5 (83.33)	1 (16.67)	0.593
	Diuretic (*N* = 13)	12 (92.31)	1 (7.69)	0.096
	ARB2 (*N* = 1)	1 (100)	0 (0.00)	0.552
	ACEI+CCB (*N* = 1)	1 (100)	0 (0.00)	0.552
	ACEI+diuretic (*N* = 6)	6 (100)	0 (0.00)	0.130
	ARB2+diuretic (*N* = 2)	1 (50.00)	1 (50.00)	0.425

*Note:* The duration of hypertensive medications varied from 1 to 22 years. MDR: resistant to at least three antibiotics. Non‐MDR: resistant to less than three antibiotics.

Abbreviations: ACEI, angiotensin‐converting enzyme inhibitor; ARB2, Angiotensin II receptor blocker; CCB, calcium channel blocker; MDR, multidrug resistance; MSSA, methicillin‐sensitive *Staphylococcus aureus*; MRSA, methicillin‐resistant *Staphylococcus aureus*; *N*, number of *Staphylococcus aureus* isolates.

### 3.7. Impact of Antihypertensive Treatment on the Resistance Profile of *Staphylococcus aureus*

**Figure 4:**
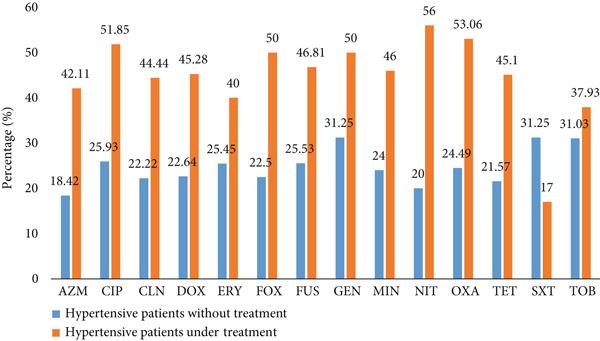
Resistance profile of antibiotics tested on *S. aureus* in relation to the antihypertensive treatment. GEN, gentamicin; TOB, tobramycin; TET, tetracycline; CLN, clindamycin; ERY, erythromycin; FUS, fusidic acid; MIN, minocycline; DOX, doxycycline; OXA, oxacillin; CIP, ciprofloxacin; NIT, nitrofurantoin; AZM, azithromycin.

Figure 4 illustrates the frequency of antibiotic resistance in hypertensive patients according to antihypertensive treatment status. The resistance rates were higher in treated hypertensive patients compared to untreated hypertensive patients for several antibiotics, including azithromycin (42.11% vs.18.42%), ciprofloxacin (51.85% vs. 25.93%), clindamycin (44.44% vs. 25.93%), doxycycline (45.28% vs. 22.64%), erythromycin (40.00% vs. 25.45%), cefoxitin (50.00% vs. 22.53%), fusidic acid (46.81% vs. 31.25%), gentamicin (50.00% vs. 31.25%), minocycline (46.00% vs. 24.00%), nitrofurantoin (56.00% vs. 20.00%), oxacillin (53.06% vs. 24.49), and tetracycline (45.10% vs. 21.57%). In contrast, the resistance rate to cotrimoxazole was lower in treated hypertensive patients compared to untreated hypertensive patients (17.00% vs. 31.25%).

### 3.8. Association Between Antibiotic Resistance Tested on *S. aureus* and Hypertension Status

Table 7 shows the association between antibiotic resistance phenotypes of *S. aureus* and hypertension status. A significant association was observed between hypertension and *S. aureus* resistance to oxacillin (OR = 8.29; 95% CI: 2.39–28.66; *p* ~ 0.001) and cotrimoxazole (OR = 6.07; 95% CI: 1.88–19.53; *p* = 0.001). Additionally, *S. aureus* isolates from hypertensive patients were more likely to be resistant to several antibiotics, including ciprofoxacin (OR = 2.70, *p* = 0.073), cefoxitin (OR = 2.25, *p* = 0.120), fusidic acid (OR = 2.90, *p* = 0.053), gentamicin (OR = 2.88, *p* = 0.120), minocycline (OR = 2.33, *p* = 0.143), and nitrofurantoin (OR = 2.24, *p* = 0.148).

**Table 7:** Association between antibiotic resistance phenotype and hypertension.

**Antibiotics**	**Hypertension**	**Nonhypertension**	**p** **value**
	**Odds ratio (95% CI)**	**Odds ratio (95% CI)**	
Azithromycin	0.61 (0.21–1.74)	1.63 (0.57–4.64)	0.358
Ciprofloxacin	2.70 (0.89–8.17)	0.36 (0.12–1.11)	0.073
Clindamycin	1.42 (0.39–5.13)	0.70 (0.19–2.51)	0.583
Doxycycline	1.81 (0.52–6.23)	0.55 (0.16–1.89)	0.339
Erythromycin	1.08 (0.28–4.16)	0.92 (0.23–3.55)	0.908
Cefoxitin	2.25 (0.80–6.37)	0.44 (0.15–1.24)	0.120
Fusidic acid	2.90 (0.96–8.76)	0.34 (0.11–1.03)	0.053
Gentamicin	2.88 (0.72–11.44)	0.34 (0.08–1.37)	0.120
Minocycline	2.33 (0.73–7.38)	0.42 (0.13–1.35)	0.143
Nitrofurantoin	2.24 (0.74–6.79)	0.44 (0.14–1.37)	0.148
Oxacillin	8.29 (2.39–28.66)^a^	0.12 (0.03–0.41)	~0.001^a^
Cotrimoxazole	6.07 (1.88–19.53)^a^	0.16 (0.05–0.59)	0.001^a^
Tetracycline	1.33 (0.40–4.36)	0.75 (0.22–2.45)	0.633
Tobramycin	1.35 (0.48–3.78)	0.73 (0.26–2.07)	0.564

*Note:* The *p* value is given at a 95% confidence interval and is significant at < 0.05. Hypertension was characterized by SBP ≥ 140 mmHg and DBP ≥ 80 mmHg.

Abbreviation: CI, confidence interval.

^a^Positive correlation between antibiotic resistance and hypertension.

## 4. Discussion

In our study, hypertension was more prevalent among women than men, which contrasts with findings from Princewel et al. [30]. Notably, widowed participants were more likely to have hypertension, whereas single participants were less affected. This finding is consistent with Segawa and his coworker′s study, which highlighted the association between hypertension and demographic factors such as gender and marital status [31]. Furthermore, participants with a primary level of education were more likely to suffer from hypertension compared to those with a higher education level, likely due to limited knowledge about hypertension risk factors and management [32].

The prevalence of *S. aureus* infection was 12.74%, which is lower than previously reported in Ethiopia (28.6%) [16] and in a study conducted at Yaounde Central Hospital, Cameroon (90.56%) [33]. However, *S. aureus* infection was more frequent in hypertensive participants compared to nonhypertensive participants, potentially due to gut microbiota dysbiosis and compromised immune function associated with hypertension [13, 34]. Interestingly, *S. aureus* was more commonly isolated in women than men, which contrasts with Marbou and Kuete′s study [26] but supports that of Odetoyin et al. [35]. Furthermore, the distribution of *S. aureus* according to antihypertensive medication revealed that patients taking CCB had the highest frequency of *S. aureus* infections. Moreover, participants who had taken CCB were more likely to be infected with MRSA than MSSA, although users of other antihypertensive drug classes also exhibited higher MRSA frequencies than MSSA. These findings are consistent with previous studies that associated ACEI and angiotensin receptor blockers (ARBs) with MRSA infections [13, 14]. This suggests that certain antihypertensive medications may increase susceptibility to *S. aureus* infections, particularly MRSA, potentially due to their impact on the immune system or gut microbiota. It has been shown that antihypertensive drugs may affect the immune response by modulating cytokine production and altering the function of immune cells [13, 36]. This finding highlights the need for further research on the relationship between antihypertensive drugs and *S. aureus* infections and the potential implications for patient care.

Bacterial infections remain a significant global health concern, with antibiotic resistance being a major contributor to treatment failure [37–39]. Antimicrobial susceptibility testing revealed a significantly higher resistance of *S. aureus* to fusidic acid, cotrimoxazole, and oxacillin antibiotics in hypertensive patients compared to nonhypertensive patients. This trend is also observed for minocycline and cefoxitin antibiotics, with higher resistance rates in hypertensive patients. High resistance to oxacillin and cefoxitin is likely due to the expression of Penicillin‐Binding Protein 2a (PBP‐2*α*) [19]. The resistance of *S. aureus* to fusidic acid, cotrimoxazole, and minocycline can be attributed to several molecular mechanisms. Mutations in the *fusA* gene are associated with resistance to fusidic acid, while mutations in the *folP* and *dhfr* genes contribute to resistance to cotrimoxazole. The acquisition of plasmids or resistance genes is also a common mechanism of resistance to cotrimoxazole. Regarding minocycline, active efflux and mutations in genes encoding ribosomal proteins are key mechanisms of resistance [40–42]. Interestingly, our study found a correlation between hypertension and *S. aureus* antibiotic resistance, which may partly explain the higher resistance rates observed in hypertensive patients.

Our study shows that MRSA frequency was significantly higher in hypertensive participants compared to nonhypertensive participants (88.37% vs. 47.83%). This rate is higher than previous studies conducted in Cameroon [31, 35], suggesting a rapid increase in MRSA. Globally, MRSA rates vary widely [43]. The high frequency of MRSA in hypertensive patients can be attributed to several factors. Firstly, hypertensive patients often have comorbidities such as diabetes and cardiovascular disease, which can make them more vulnerable to infections, particularly *S. aureus* infections [44, 45]. Secondly, hypertensive individuals are at risk of intestinal dysbiosis, characterized by reduced bacterial biodiversity, which increases bacterial susceptibility to bacterial infections [8, 11, 12]. Finally, hypertensive patients often have frequent contacts with healthcare facilities, which can increase their risk of exposure to resistant bacteria like MRSA [46, 47].

Additionally, all MRSA isolates were MDR, with a significant difference compared to MSSA. This finding is consistent with those obtained by Kengne et al. and Bauer et al. [20, 25]. The high resistance of MRSA can be attributed to the fact that MRSA acquires antibiotic resistance through a mobile genetic element called *SCCmec*, which contains the *mecA* gene [48]. This gene encodes a PBP‐2*α* that confers resistance to most *β*‐lactam antibiotics. MRSA can also develop resistance to other antibiotics through various mechanisms, making them MDR [49].

In this study, MDR was defined as resistance to three or more antibiotic classes [34]. Notably, our findings revealed that all *S. aureus* isolates from hypertensive participants exhibited MDR, significantly surpassing the rate observed in nonhypertensive participants. These results are consistent with those of Odetoyin et al. [35], who reported similarly high MDR rates in hypertensive patients. The high MDR prevalence in our study may be attributed to the inadequate and excessive use of antibiotics without medical supervision. Furthermore, two key mechanisms may underlie this phenomenon: the accumulation of multiple resistance genes within a single cell, often facilitated by resistance plasmids, and the upregulation of genes encoding multidrug efflux pumps, which can expel a broad range of antibiotics [50]. The elevated prevalence of MDR among hypertensive patients receiving antihypertensive therapy may be attributed to the immunomodulatory effects of certain antihypertensive agents, which potentially increase susceptibility to *Staphylococcus aureus* infections. This compromised immune response may impair the ability of hypertensive individuals to effectively combat MDR bacterial pathogens [13, 51].

The findings presented herein hold significant public health implications, as the relationship between antibiotic resistance in *S. aureus* and hypertension remains poorly understood, despite extensive research on the susceptibility of hypertensive patients to MDR bacterial infections. While this study provides valuable insights into the association between hypertension, antihypertensive medication, and antibiotic resistance in *S. aureus*, several limitations must be acknowledged. Notably, the study′s design did not account for variables such as duration of hypertension, duration of antihypertensive treatment, prior antibiotic exposure, and history of *S. aureus* resistance, which may have impacted the results. Furthermore, the data may not be representative of the entire population, and the small sample size may limit the generalizability of the findings. Additionally, the identification of *S. aureus* was based solely on phenotypic methods, which may not provide unequivocal identification. The lack of adjustment for potential confounding factors, such as age and comorbidities, is another significant limitation. Moreover, the study did not include genetic diversity data of *S. aureus*, which could have provided further insights into the mechanisms of antibiotic resistance and the transmission dynamics of *S. aureus*. Notwithstanding these limitations, we believe that this study provides crucial information on the intestinal carriage of MDR MRSA in hypertensive patients and sheds light on the relationship between hypertension and the emergence of resistance in *S. aureus*.

## 5. Conclusion

This study reveals that *S. aureus* exhibits high resistance to many of the common antimicrobials used in Cameroon. This study suggests that hypertensive patients harbor MRSA strains in their intestines, with significant differences compared to nonhypertensive participants. The higher resistance of *S. aureus* to oxacillin, fusidic acid, cotrimoxazole, minocycline, doxycycline, nitrofurantoin, and cefoxitin was associated with hypertension. The prevalence of MDR *S. aureus* was higher in hypertensive participants than in nonhypertensive participants. *S. aureus* resistance to antibiotics was higher in hypertensive participants who were on treatment than in untreated hypertensive participants. The need for appropriate antimicrobial use to tackle, or at least limit, the spread of resistance is suggested in the care of hypertensive patients with enteric infection caused by *S. aureus*. Further research that focuses on identifying dynamics promoting resistance, identifying high‐risk strains, and the molecular genetic basis of resistance is needed. We suggest that clinicians should focus more on intestinal colonization by MDR *S. aureus* in hypertensive patients and take into account the relationship between hypertension and resistance pattern when initiating antimicrobial therapy.

## Ethics Statement

All procedures involving human participants or human‐derived samples were conducted in accordance with institutional guidelines and approved by the Institutional Ethics Committee for Research on Human Health of the University of Douala, Littoral, Cameroon (Reference No. 3031CEI‐UDo/06/2022/T).

## Consent

Each participant gave written and informed consent for voluntary participation.

## Disclosure

All the authors read and approved the final manuscript.

## Conflicts of Interest

The authors declare no conflicts of interest.

## Author Contributions

O.D.T. carried out the study and helped in writing the manuscript. O.D.T., M.F.K., and B.S.T.D. drafted the original manuscript. O.D.T. performed the statistical analysis. A.T.M. and V.K. designed and supervised the study. A.T.M. and V.K. edited the final manuscript.

## Funding

No funding was received for this manuscript.

## Supporting information


**Supporting Information** Additional supporting information can be found online in the Supporting Information section. Supporting information file (.PDF): Table S1: Participants and their features. Table S2: Patients, hypertensive status, and treatments. Table S3: Patients, *S. aureus* infection, and antibiogram.

## Data Availability

The data used to support the findings of this study are included in the supporting information file.
