# Supplementary material for: Fecal Carriage of Multidrug‐Resistant Staphylococcus aureus in Hypertensive Patients at the Douala Laquintinie Hospital: Prevalence and Resistance Patterns
Source: Biomed Res Int. 2025 Nov 26;2025:8076503. doi: 10.1155/bmri/8076503 (PMC12649821; doi:10.1155/bmri/8076503)
Supplement: Supplementary file 1 — Supporting Information Additional supporting information can be found online in the Supporting Information section. Supporting information file (.PDF): Table S1: Participants and their features. Table S2: Patients, hypertensive status, and treatments. Table S3: Patients, S. aureus infection, and antibiogram. [file BMRI-2025-8076503-s001.docx]

Fecal Carriage of Multidrug-Resistant *Staphylococcus aureus* in Hypertensive Patients at the Douala Laquintinie Hospital: Prevalence and Resistance Patterns

*Ornella Djiolieu Tsobeng^a^, Armelle T. Mbaveng^a^*, Michael F. Kengne^a^, Ballue S. T. Dadjo^a^, Victor Kuete^a**^*

*^a^Department of Biochemistry, Faculty of Science, Université de Dschang, Dschang, Cameroon*

***Corresponding authors:***

**Email:* [*armbatsa@yahoo.fr*](mailto:armbatsa@yahoo.fr)*; ORCID:* [*https://orcid.org/0000-0003-4178-4967*](https://orcid.org/0000-0003-4178-4967) *(Armelle T. Mbaveng)*

** *Email:* [*kuetevictor@yahoo.fr*](mailto:kuetevictor@yahoo.fr)*; ORCID:* [*http://orcid.org/0000-0002-1070-1236*](http://orcid.org/0000-0002-1070-1236) *(Victor Kuete)*

***Other authors emails:***

*Email: tsobengornella@yahoo.com (Ornella Djiolieu Tsobeng)*

*Email:* [*fmkengne@yahoo.com*](mailto:fmkengne@yahoo.com) *(Michael F. Kengne)*

*Email:* [*ballueserges@gmail.com*](mailto:ballueserges@gmail.com) *(Ballue S. T. Dadjo)*

**Suppl Table 1. Participants and their features**

| Patient Codes | Sex | Ages (years) | Age groups (years) | SBP (mmHg) | DBP (mmHg) | Pulsation (bpm) | Education Level | Matrimonial status | Family history of hypertension |
| --- | --- | --- | --- | --- | --- | --- | --- | --- | --- |
| D201 | Female | 25 | 20-40 | 152 | 95 | 85 | Higher education | Single | No |
| T002 | Male | 83 | ≥80 | 154 | 75 | 75 | Secondary school | Married | Yes |
| T003 | Male | 62 | 60-80 | 172 | 108 | 89 | Elementary school | Married | No |
| T004 | Male | 63 | 60-80 | 177 | 104 | 111 | Secondary school | Single | Yes |
| D202 | Male | 43 | 40-60 | 138 | 84 | 72 | Higher education | Married | No |
| T006 | Male | 79 | 60-80 | 130 | 86 | 74 | Secondary school | Widow(er) | Yes |
| T007 | Male | 53 | 40-60 | 144 | 108 | 98 | Secondary school | Married | Yes |
| D203 | Female | 45 | 40-60 | 113 | 66 | 110 | Secondary school | Single | No |
| D204 | Male | 32 | 20-40 | 122 | 67 | 70 | Higher education | Single | No |
| D205 | Male | 21 | 20-40 | 105 | 68 | 67 | Higher education | Single | No |
| T011 | Female | 72 | 60-80 | 161 | 110 | 92 | Elementary school | Married | Yes |
| T012 | Female | 51 | 40-60 | 172 | 107 | 94 | Secondary school | Widow(er) | Yes |
| T013 | Female | 58 | 40-60 | 102 | 103 | 78 | Secondary school | Single | Yes |
| T014 | Male | 47 | 40-60 | 154 | 114 | 66 | Secondary school | Married | No |
| T015 | Male | 77 | 60-80 | 169 | 103 | 96 | Elementary school | Married | Yes |
| T016 | Female | 88 | ≥80 | 157 | 94 | 98 | Elementary school | Married | No |
| T017 | Male | 44 | 40-60 | 172 | 126 | 100 | Secondary school | Married | No |
| T018 | Female | 52 | 40-60 | 173 | 99 | 62 | Secondary school | Married | No |
| T019 | Female | 79 | 60-80 | 190 | 121 | 93 | Illiterate | Widow(er) | No |
| T020 | Female | 56 | 40-60 | 170 | 92 | 60 | Secondary school | Married | Yes |
| T021 | Female | 54 | 40-60 | 178 | 104 | 101 | Secondary school | Widow(er) | Yes |
| T022 | Female | 49 | 40-60 | 165 | 100 | 98 | Secondary school | Married | Yes |
| T023 | Male | 74 | 60-80 | 156 | 85 | 87 | Secondary school | Married | Yes |
| T024 | Male | 69 | 60-80 | 165 | 96 | 80 | Secondary school | Widow(er) | Yes |
| T025 | Female | 63 | 60-80 | 134 | 87 | 68 | Elementary school | Married | Yes |
| T026 | Male | 66 | 60-80 | 150 | 90 | 83 | Secondary school | Married | Yes |
| T027 | Female | 62 | 60-80 | 136 | 96 | 70 | Elementary school | Married | Yes |
| T028 | Female | 67 | 60-80 | 151 | 99 | 88 | Secondary school | Married | No |
| D206 | Female | 24 | 20-40 | 123 | 70 | 84 | Higher education | Married | Yes |
| T030 | Male | 61 | 60-80 | 203 | 100 | 42 | Secondary school | Married | No |
| T031 | Male | 48 | 40-60 | 155 | 105 | 119 | Secondary school | Married | No |
| T032 | Male | 60 | 60-80 | 159 | 106 | 109 | Secondary school | Married | No |
| D207 | Female | 39 | 20-40 | 128 | 72 | 80 | Secondary school | Married | No |
| D208 | Male | 31 | 20-40 | 138 | 83 | 74 | Higher education | Single | Yes |
| D209 | Male | 59 | 40-60 | 129 | 80 | 84 | Higher education | Married | No |
| D210 | Female | 34 | 20-40 | 113 | 97 | 91 | Higher education | Married | No |
| T037 | Female | 58 | 40-60 | 165 | 104 | 89 | Secondary school | Married | Yes |
| T038 | Female | 72 | 60-80 | 223 | 152 | 88 | Illiterate | Widow(er) | No |
| T039 | Male | 33 | 20-40 | 204 | 135 | 101 | Elementary school | Single | No |
| T040 | Female | 78 | 60-80 | 137 | 84 | 86 | Higher education | Widow(er) | No |
| T041 | Female | 52 | 40-60 | 177 | 127 | 100 | Secondary school | Married | No |
| T042 | Male | 45 | 40-60 | 189 | 138 | 74 | Elementary school | Married | Yes |
| T043 | Female | 61 | 60-80 | 162 | 92 | 54 | Secondary school | Married | No |
| T044 | Female | 83 | ≥80 | 160 | 77 | 69 | Illiterate | Single | Yes |
| T045 | Male | 72 | 60-80 | 159 | 76 | 84 | Secondary school | Married | No |
| T046 | Male | 61 | 60-80 | 112 | 64 | 79 | Secondary school | Married | Yes |
| T047 | Male | 34 | 20-40 | 203 | 106 | 99 | Higher education | Single | No |
| T048 | Male | 38 | 20-40 | 135 | 79 | 69 | Higher education | Married | Yes |
| T049 | Female | 73 | 60-80 | 206 | 137 | 154 | Secondary school | Widow(er) | Yes |
| T050 | Female | 60 | 60-80 | 175 | 120 | 90 | Secondary school | Married | Yes |
| T051 | Female | 65 | 60-80 | 161 | 84 | 90 | Secondary school | Married | Yes |
| T052 | Male | 71 | 60-80 | 142 | 76 | 86 | Secondary school | Married | No |
| T053 | Female | 54 | 40-60 | 118 | 80 | 100 | Elementary school | Widow(er) | Yes |
| T054 | Male | 87 | ≥80 | 149 | 81 | 81 | Elementary school | Widow(er) | No |
| D211 | Male | 49 | 40-60 | 104 | 65 | 78 | Illiterate | Married | No |
| D212 | Female | 52 | 40-60 | 142 | 98 | 74 | Secondary school | Married | Yes |
| T057 | Female | 77 | 60-80 | 146 | 98 | 89 | Illiterate | Widow(er) | Yes |
| T058 | Female | 33 | 20-40 | 176 | 131 | 99 | Secondary school | Single | Yes |
| T059 | Female | 75 | 60-80 | 203 | 154 | 150 | Elementary school | Widow(er) | Yes |
| T060 | Male | 61 | 60-80 | 140 | 78 | 80 | Secondary school | Married | Yes |
| T061 | Female | 69 | 60-80 | 173 | 112 | 111 | Elementary school | Widow(er) | Yes |
| T062 | Male | 44 | 40-60 | 177 | 115 | 125 | Secondary school | Single | Yes |
| T063 | Female | 78 | 60-80 | 126 | 94 | 104 | Elementary school | Married | No |
| T064 | Female | 47 | 40-60 | 190 | 101 | 79 | Elementary school | Single | No |
| T065 | Female | 72 | 60-80 | 245 | 134 | 80 | Illiterate | Widow(er) | No |
| T066 | Male | 72 | 60-80 | 160 | 88 | 51 | Elementary school | Married | No |
| T067 | Male | 70 | 60-80 | 150 | 95 | 106 | Secondary school | Married | No |
| T068 | Female | 80 | ≥80 | 208 | 167 | 122 | Illiterate | Widow(er) | No |
| T069 | Male | 63 | 60-80 | 163 | 90 | 86 | Secondary school | Married | Yes |
| T070 | Female | 83 | ≥80 | 123 | 91 | 90 | Elementary school | Widow(er) | No |
| T071 | Female | 59 | 40-60 | 185 | 113 | 78 | Elementary school | Married | No |
| T072 | Male | 72 | 60-80 | 138 | 77 | 66 | Illiterate | Married | Yes |
| T073 | Male | 60 | 60-80 | 122 | 88 | 107 | Secondary school | Married | No |
| T074 | Female | 48 | 40-60 | 231 | 159 | 117 | Secondary school | Married | No |
| D213 | Female | 23 | 20-40 | 129 | 81 | 78 | Higher education | Single | No |
| T076 | Male | 54 | 40-60 | 175 | 105 | 88 | Secondary school | Married | Yes |
| T077 | Male | 62 | 60-80 | 160 | 101 | 72 | Elementary school | Married | No |
| T078 | Female | 72 | 60-80 | 197 | 102 | 60 | Illiterate | Widow(er) | Yes |
| T079 | Female | 38 | 20-40 | 182 | 116 | 88 | Elementary school | Single | Yes |
| T080 | Female | 75 | 60-80 | 140 | 68 | 94 | Elementary school | Widow(er) | No |
| T081 | Male | 57 | 40-60 | 183 | 96 | 85 | Elementary school | Married | No |
| T082 | Female | 70 | 60-80 | 139 | 98 | 98 | Illiterate | Widow(er) | No |
| T083 | Male | 56 | 40-60 | 179 | 119 | 91 | Secondary school | Married | Yes |
| T084 | Female | 57 | 40-60 | 197 | 136 | 78 | Illiterate | Married | Yes |
| T085 | Female | 70 | 60-80 | 166 | 101 | 65 | Elementary school | Married | Yes |
| T086 | Female | 71 | 60-80 | 111 | 66 | 89 | Secondary school | Widow(er) | No |
| T087 | Female | 72 | 60-80 | 145 | 72 | 107 | Illiterate | Widow(er) | No |
| T088 | Male | 87 | ≥80 | 201 | 110 | 133 | Illiterate | Widow(er) | No |
| T089 | Female | 63 | 60-80 | 188 | 123 | 138 | Secondary school | Widow(er) | Yes |
| T090 | Male | 56 | 40-60 | 183 | 116 | 136 | Secondary school | Single | Yes |
| T091 | Male | 40 | 40-60 | 192 | 146 | 76 | Elementary school | Married | No |
| T092 | Female | 54 | 40-60 | 214 | 138 | 127 | Higher education | Single | Yes |
| D214 | Female | 45 | 40-60 | 124 | 85 | 100 | Secondary school | Married | Yes |
| T094 | Female | 73 | 60-80 | 128 | 88 | 82 | Elementary school | Married | Yes |
| T095 | Female | 58 | 40-60 | 175 | 108 | 100 | Secondary school | Married | Yes |
| T096 | Female | 57 | 40-60 | 153 | 88 | 69 | Secondary school | Widow(er) | Yes |
| T097 | Male | 67 | 60-80 | 163 | 101 | 67 | Elementary school | Married | No |
| T098 | Male | 57 | 40-60 | 148 | 96 | 96 | Elementary school | Married | Yes |
| T099 | Male | 57 | 40-60 | 173 | 108 | 79 | Elementary school | Married | No |
| T100 | Male | 87 | ≥80 | 155 | 87 | 98 | Illiterate | Married | No |
| T101 | Female | 80 | ≥80 | 173 | 140 | 100 | Illiterate | Widow(er) | Yes |
| T102 | Female | 64 | 60-80 | 156 | 73 | 76 | Elementary school | Married | No |
| T103 | Male | 55 | 40-60 | 152 | 99 | 78 | Secondary school | Single | Yes |
| T104 | Male | 65 | 60-80 | 183 | 109 | 87 | Elementary school | Married | Yes |
| T105 | Male | 56 | 40-60 | 180 | 90 | 75 | Elementary school | Married | Yes |
| T106 | Female | 55 | 40-60 | 168 | 97 | 80 | Higher education | Widow(er) | Yes |
| T107 | Male | 59 | 40-60 | 159 | 110 | 65 | Elementary school | Married | No |
| T108 | Male | 57 | 40-60 | 170 | 102 | 99 | Secondary school | Married | Yes |
| T109 | Female | 65 | 60-80 | 160 | 101 | 102 | Secondary school | Widow(er) | Yes |
| T110 | Female | 62 | 60-80 | 187 | 99 | 70 | Secondary school | Married | No |
| T111 | Male | 32 | 20-40 | 145 | 118 | 64 | Secondary school | Single | Yes |
| T112 | Male | 52 | 40-60 | 192 | 109 | 79 | Illiterate | Married | No |
| T113 | Male | 51 | 40-60 | 177 | 112 | 80 | Secondary school | Married | Yes |
| T114 | Male | 73 | 60-80 | 148 | 97 | 74 | Secondary school | Married | Yes |
| T115 | Female | 68 | 60-80 | 196 | 111 | 102 | Elementary school | Married | Yes |
| T116 | Male | 63 | 60-80 | 185 | 79 | 76 | Elementary school | Married | Yes |
| T117 | Female | 62 | 60-80 | 169 | 119 | 95 | Elementary school | Widow(er) | Yes |
| T118 | Female | 51 | 40-60 | 183 | 110 | 96 | Secondary school | Married | Yes |
| T119 | Female | 52 | 40-60 | 171 | 103 | 105 | Secondary school | Married | Yes |
| T120 | Female | 40 | 40-60 | 203 | 135 | 100 | Secondary school | Married | No |
| T121 | Male | 58 | 40-60 | 149 | 96 | 95 | Higher education | Married | Yes |
| T122 | Male | 53 | 40-60 | 193 | 123 | 89 | Secondary school | Married | Yes |
| T123 | Male | 60 | 60-80 | 136 | 118 | 80 | Secondary school | Married | No |
| T124 | Male | 55 | 40-60 | 181 | 93 | 83 | Secondary school | Married | Yes |
| T125 | Male | 68 | 60-80 | 198 | 74 | 74 | Elementary school | Married | No |
| T126 | Male | 49 | 40-60 | 141 | 104 | 100 | Higher education | Married | Yes |
| T127 | Male | 52 | 40-60 | 213 | 125 | 99 | Secondary school | Married | Yes |
| T128 | Female | 56 | 40-60 | 166 | 110 | 80 | Secondary school | Widow(er) | Yes |
| T129 | Female | 40 | 40-60 | 149 | 110 | 79 | Secondary school | Married | Yes |
| T130 | Female | 52 | 40-60 | 145 | 98 | 81 | Secondary school | Married | Yes |
| T131 | Female | 35 | 20-40 | 193 | 122 | 97 | Secondary school | Married | Yes |
| T132 | Male | 68 | 60-80 | 175 | 106 | 77 | Elementary school | Married | No |
| T133 | Male | 62 | 60-80 | 178 | 121 | 86 | Secondary school | Married | Yes |
| T134 | Female | 57 | 40-60 | 189 | 83 | 113 | Secondary school | Married | Yes |
| T135 | Female | 68 | 60-80 | 142 | 104 | 79 | Secondary school | Married | Yes |
| T136 | Male | 52 | 40-60 | 175 | 105 | 89 | Higher education | Married | Yes |
| T137 | Female | 46 | 40-60 | 151 | 95 | 85 | Secondary school | Married | Yes |
| T138 | Male | 33 | 20-40 | 149 | 102 | 100 | Secondary school | Single | Yes |
| T139 | Male | 63 | 60-80 | 147 | 96 | 89 | Secondary school | Married | Yes |
| T140 | Female | 74 | 60-80 | 151 | 88 | 98 | Illiterate | Widow(er) | No |
| T141 | Female | 50 | 40-60 | 194 | 111 | 99 | Higher education | Single | Yes |
| T142 | Female | 62 | 60-80 | 160 | 99 | 87 | Higher education | Married | Yes |
| T143 | Male | 52 | 40-60 | 156 | 94 | 60 | Secondary school | Married | Yes |
| T144 | Female | 47 | 40-60 | 159 | 102 | 89 | Secondary school | Married | Yes |
| T145 | Female | 78 | 60-80 | 149 | 98 | 79 | Elementary school | Widow(er) | No |
| T146 | Male | 60 | 60-80 | 147 | 94 | 98 | Secondary school | Married | Yes |
| T147 | Male | 48 | 40-60 | 147 | 101 | 103 | Secondary school | Married | Yes |
| T148 | Male | 53 | 40-60 | 128 | 79 | 107 | Secondary school | Widow(er) | No |
| T149 | Female | 66 | 60-80 | 189 | 117 | 62 | Elementary school | Widow(er) | Yes |
| T150 | Female | 51 | 40-60 | 133 | 100 | 78 | Secondary school | Married | Yes |
| T151 | Female | 46 | 40-60 | 178 | 107 | 89 | Secondary school | Married | Yes |
| T152 | Female | 53 | 40-60 | 140 | 98 | 79 | Secondary school | Married | Yes |
| T153 | Female | 45 | 40-60 | 149 | 96 | 88 | Secondary school | Married | Yes |
| T154 | Female | 37 | 20-40 | 163 | 115 | 65 | Secondary school | Single | Yes |
| T155 | Female | 68 | 60-80 | 157 | 107 | 75 | Secondary school | Married | Yes |
| T156 | Male | 70 | 60-80 | 219 | 116 | 84 | Secondary school | Married | Yes |
| T157 | Female | 56 | 40-60 | 204 | 127 | 102 | Secondary school | Married | No |
| T158 | Male | 48 | 40-60 | 141 | 107 | 89 | Secondary school | Single | Yes |
| T159 | Female | 64 | 60-80 | 149 | 87 | 79 | Secondary school | Married | No |
| T160 | Female | 69 | 60-80 | 181 | 85 | 78 | Illiterate | Widow(er) | Yes |
| T161 | Male | 52 | 40-60 | 199 | 128 | 90 | Secondary school | Married | Yes |
| T162 | Male | 52 | 40-60 | 210 | 125 | 89 | Secondary school | Married | Yes |
| T163 | Male | 63 | 60-80 | 165 | 101 | 96 | Elementary school | Single | Yes |
| T164 | Female | 53 | 40-60 | 162 | 77 | 77 | Higher education | Single | No |
| T165 | Male | 61 | 60-80 | 200 | 105 | 109 | Higher education | Married | Yes |
| T166 | Male | 62 | 60-80 | 146 | 88 | 65 | Secondary school | Married | Yes |
| T167 | Female | 68 | 60-80 | 162 | 94 | 71 | Secondary school | Widow(er) | Yes |
| T168 | Female | 72 | 60-80 | 132 | 86 | 94 | Secondary school | Married | No |
| T169 | Male | 69 | 60-80 | 182 | 89 | 75 | Secondary school | Married | Yes |
| T170 | Male | 53 | 40-60 | 150 | 90 | 79 | Secondary school | Married | No |
| T171 | Female | 79 | 60-80 | 145 | 88 | 99 | Illiterate | Widow(er) | No |
| T172 | Female | 71 | 60-80 | 126 | 83 | 84 | Secondary school | Married | Yes |
| T173 | Female | 87 | ≥80 | 255 | 95 | 73 | Illiterate | Widow(er) | Yes |
| T174 | Female | 60 | 60-80 | 159 | 94 | 101 | Secondary school | Widow(er) | Yes |
| T175 | Female | 69 | 60-80 | 168 | 98 | 84 | Secondary school | Widow(er) | No |
| T176 | Female | 40 | 40-60 | 214 | 118 | 80 | Higher education | Single | Yes |
| T177 | Male | 68 | 60-80 | 152 | 104 | 81 | Secondary school | Married | No |
| T178 | Female | 65 | 60-80 | 214 | 177 | 110 | Elementary school | Married | Yes |
| T179 | Female | 64 | 60-80 | 149 | 83 | 96 | Secondary school | Widow(er) | No |
| T180 | Female | 56 | 40-60 | 155 | 87 | 81 | Elementary school | Married | Yes |
| T181 | Female | 38 | 20-40 | 195 | 119 | 84 | Secondary school | Single | Yes |
| T182 | Female | 52 | 40-60 | 149 | 90 | 89 | Secondary school | Single | Yes |
| T183 | Female | 41 | 40-60 | 128 | 84 | 92 | Elementary school | Married | Yes |
| T184 | Female | 78 | 60-80 | 188 | 116 | 81 | Secondary school | Married | Yes |
| T185 | Male | 46 | 40-60 | 146 | 94 | 78 | Secondary school | Married | Yes |
| T186 | Male | 68 | 60-80 | 162 | 102 | 89 | Secondary school | Widow(er) | No |
| T187 | Female | 72 | 60-80 | 161 | 88 | 103 | Illiterate | Widow(er) | No |
| T188 | Female | 66 | 60-80 | 145 | 86 | 79 | Secondary school | Married | No |
| T189 | Female | 50 | 40-60 | 185 | 115 | 71 | Illiterate | Married | No |
| T190 | Male | 79 | 60-80 | 135 | 83 | 70 | Secondary school | Married | No |
| T191 | Male | 52 | 40-60 | 140 | 104 | 111 | Secondary school | Married | Yes |
| T192 | Female | 42 | 40-60 | 161 | 100 | 88 | Secondary school | Married | No |
| T193 | Male | 44 | 40-60 | 175 | 115 | 79 | Secondary school | Single | Yes |
| T194 | Female | 49 | 40-60 | 138 | 83 | 77 | Higher education | Married | Yes |
| T195 | Male | 40 | 40-60 | 151 | 91 | 80 | Secondary school | Single | No |
| D214 | Female | 59 | 40-60 | 123 | 88 | 87 | Secondary school | Married | No |
| T197 | Male | 53 | 40-60 | 151 | 83 | 70 | Elementary school | Married | No |
| T198 | Female | 64 | 60-80 | 180 | 65 | 76 | Secondary school | Widow(er) | Yes |
| T199 | Female | 66 | 60-80 | 129 | 80 | 66 | Secondary school | Widow(er) | Yes |
| T200 | Female | 61 | 60-80 | 161 | 100 | 90 | Secondary school | Married | Yes |
| T201 | Female | 66 | 60-80 | 131 | 76 | 71 | Elementary school | Widow(er) | Yes |
| T202 | Female | 59 | 40-60 | 162 | 104 | 82 | Secondary school | Married | Yes |
| T203 | Female | 54 | 40-60 | 121 | 80 | 74 | Secondary school | Widow(er) | Yes |
| T204 | Female | 50 | 40-60 | 198 | 93 | 63 | Secondary school | Single | Yes |
| T205 | Female | 67 | 60-80 | 183 | 81 | 103 | Elementary school | Widow(er) | Yes |
| T206 | Female | 69 | 60-80 | 145 | 81 | 80 | Elementary school | Widow(er) | Yes |
| T207 | Male | 60 | 60-80 | 177 | 92 | 80 | Higher education | Married | Yes |
| T208 | Female | 59 | 40-60 | 187 | 107 | 104 | Elementary school | Married | Yes |
| T209 | Male | 57 | 40-60 | 152 | 97 | 58 | Higher education | Married | Yes |
| T210 | Male | 59 | 40-60 | 156 | 58 | 53 | Higher education | Married | No |
| T211 | Male | 79 | 60-80 | 159 | 98 | 118 | Elementary school | Married | No |
| T212 | Male | 66 | 60-80 | 139 | 85 | 76 | Elementary school | Married | Yes |
| T213 | Female | 35 | 20-40 | 151 | 84 | 90 | Elementary school | Married | No |
| T214 | Male | 56 | 40-60 | 178 | 81 | 53 | Illiterate | Married | No |
| T215 | Female | 67 | 60-80 | 196 | 111 | 81 | Elementary school | Widow(er) | Yes |
| T216 | Male | 70 | 60-80 | 112 | 80 | 102 | Secondary school | Married | No |
| T217 | Female | 45 | 40-60 | 157 | 101 | 93 | Secondary school | Married | Yes |
| T218 | Male | 51 | 40-60 | 137 | 91 | 66 | Secondary school | Married | Yes |
| T219 | Male | 42 | 40-60 | 143 | 94 | 63 | Secondary school | Married | No |
| T220 | Female | 67 | 60-80 | 164 | 90 | 82 | Secondary school | Married | Yes |
| T221 | Male | 51 | 40-60 | 134 | 87 | 73 | Secondary school | Married | Yes |
| T222 | Female | 60 | 60-80 | 155 | 94 | 80 | Secondary school | Married | Yes |
| T223 | Male | 48 | 40-60 | 145 | 110 | 103 | Secondary school | Married | Yes |
| T224 | Male | 75 | 60-80 | 171 | 95 | 63 | Secondary school | Widow(er) | Yes |
| T225 | Male | 57 | 40-60 | 168 | 120 | 86 | Secondary school | Widow(er) | Yes |
| T226 | Female | 61 | 60-80 | 160 | 81 | 67 | Secondary school | Single | Yes |
| T227 | Male | 46 | 40-60 | 151 | 113 | 64 | Higher education | Married | Yes |
| T228 | Female | 54 | 40-60 | 141 | 80 | 60 | Secondary school | Married | Yes |
| T229 | Male | 74 | 60-80 | 149 | 74 | 50 | Secondary school | Married | Yes |
| T230 | Female | 61 | 60-80 | 188 | 108 | 66 | Secondary school | Single | Yes |
| T231 | Female | 51 | 40-60 | 129 | 93 | 54 | Elementary school | Married | Yes |
| T232 | Female | 52 | 40-60 | 108 | 84 | 67 | Elementary school | Single | No |
| T233 | Male | 63 | 60-80 | 156 | 83 | 82 | Elementary school | Married | No |
| T234 | Female | 36 | 20-40 | 176 | 128 | 66 | Higher education | Married | Yes |
| T235 | Female | 61 | 60-80 | 210 | 111 | 99 | Elementary school | Widow(er) | Yes |
| T236 | Male | 68 | 60-80 | 161 | 94 | 43 | Elementary school | Married | No |
| T237 | Female | 84 | ≥80 | 179 | 77 | 69 | Elementary school | Widow(er) | No |
| T238 | Female | 75 | 60-80 | 162 | 83 | 90 | Secondary school | Widow(er) | No |
| T239 | Female | 75 | 60-80 | 176 | 103 | 69 | Secondary school | Widow(er) | Yes |
| T240 | Female | 49 | 40-60 | 168 | 91 | 73 | Elementary school | Married | Yes |
| T241 | Female | 61 | 60-80 | 166 | 118 | 96 | Elementary school | Married | Yes |
| T242 | Female | 75 | 60-80 | 180 | 112 | 80 | Elementary school | Widow(er) | No |
| T243 | Female | 50 | 40-60 | 198 | 107 | 87 | Secondary school | Widow(er) | Yes |
| T244 | Male | 32 | 20-40 | 157 | 99 | 88 | Higher education | Single | Yes |
| T245 | Female | 38 | 20-40 | 129 | 80 | 75 | Higher education | Single | Yes |
| D001 | Male | 45 | 40-60 | 120 | 80 | 86 | Secondary school | Married | No |
| D002 | Female | 64 | 60-80 | 139 | 92 | 82 | Elementary school | Married | Yes |
| D003 | Female | 52 | 40-60 | 139 | 80 | 100 | Secondary school | Married | No |
| D004 | Male | 42 | 40-60 | 128 | 89 | 87 | Secondary school | Married | Yes |
| D005 | Male | 69 | 60-80 | 138 | 70 | 98 | Secondary school | Widow(er) | No |
| D006 | Female | 34 | 20-40 | 129 | 67 | 101 | Secondary school | Married | Yes |
| D007 | Male | 49 | 40-60 | 140 | 79 | 66 | Secondary school | Married | No |
| D008 | Female | 44 | 40-60 | 122 | 88 | 99 | Elementary school | Married | Yes |
| D009 | Male | 77 | 60-80 | 120 | 77 | 102 | Illiterate | Widow(er) | No |
| D011 | Male | 40 | 40-60 | 130 | 98 | 70 | Higher education | Married | Yes |
| D012 | Male | 31 | 20-40 | 133 | 77 | 110 | Secondary school | Single | No |
| D013 | Female | 38 | 20-40 | 120 | 60 | 60 | Elementary school | Married | No |
| D014 | Female | 55 | 40-60 | 110 | 89 | 77 | Secondary school | Married | No |
| D015 | Female | 41 | 40-60 | 114 | 70 | 60 | Elementary school | Married | Yes |
| D016 | Female | 20 | 20-40 | 119 | 78 | 88 | Secondary school | Single | No |
| D017 | Female | 61 | 60-80 | 121 | 67 | 74 | Elementary school | Married | No |
| D018 | Female | 23 | 20-40 | 127 | 90 | 88 | Secondary school | Single | Yes |
| D019 | Female | 53 | 40-60 | 130 | 70 | 100 | Secondary school | Married | Yes |
| D020 | Female | 34 | 20-40 | 126 | 67 | 59 | Secondary school | Single | No |
| D021 | Female | 36 | 20-40 | 122 | 77 | 81 | Secondary school | Married | No |
| D022 | Male | 20 | 20-40 | 107 | 70 | 50 | Secondary school | Single | Yes |
| D023 | Male | 28 | 20-40 | 128 | 78 | 88 | Secondary school | Single | No |
| D024 | Male | 47 | 40-60 | 138 | 90 | 79 | Secondary school | Married | Yes |
| D025 | Female | 67 | 60-80 | 139 | 79 | 70 | Illiterate | Widow(er) | No |
| D027 | Female | 40 | 40-60 | 120 | 79 | 80 | Secondary school | Married | No |
| D028 | Male | 35 | 20-40 | 130 | 90 | 70 | Secondary school | Married | No |
| D029 | Male | 34 | 20-40 | 120 | 78 | 66 | Secondary school | Married | Yes |
| D030 | Male | 72 | 60-80 | 118 | 88 | 98 | Illiterate | Widow(er) | No |
| D031 | Female | 45 | 40-60 | 128 | 77 | 75 | Secondary school | Married | Yes |
| T246 | Female | 65 | 60-80 | 132 | 73 | 73 | Secondary school | Widow(er) | No |
| D034 | Female | 38 | 20-40 | 107 | 60 | 90 | Elementary school | Married | No |
| D035 | Male | 57 | 40-60 | 127 | 78 | 69 | Secondary school | Married | No |
| D036 | Female | 20 | 20-40 | 118 | 77 | 80 | Secondary school | Single | No |
| D037 | Female | 54 | 40-60 | 110 | 70 | 97 | Secondary school | Married | No |
| D038 | Female | 79 | 60-80 | 111 | 79 | 102 | Illiterate | Widow(er) | No |
| D039 | Female | 25 | 20-40 | 114 | 69 | 79 | Higher education | Single | Yes |
| D040 | Female | 58 | 40-60 | 120 | 85 | 97 | Secondary school | Married | No |
| D041 | Male | 27 | 20-40 | 130 | 110 | 78 | Secondary school | Single | Yes |
| D042 | Female | 66 | 60-80 | 129 | 67 | 89 | Elementary school | Married | No |
| D044 | Female | 37 | 20-40 | 120 | 70 | 67 | Secondary school | Married | No |
| D045 | Female | 78 | 60-80 | 102 | 89 | 78 | Elementary school | Widow(er) | No |
| D046 | Male | 63 | 60-80 | 80 | 73 | 101 | Elementary school | Married | No |
| D047 | Male | 43 | 40-60 | 115 | 56 | 77 | Secondary school | Married | Yes |
| D048 | Female | 63 | 60-80 | 125 | 49 | 103 | Secondary school | Widow(er) | No |
| D049 | Male | 52 | 40-60 | 116 | 56 | 75 | Secondary school | Married | Yes |
| D050 | Female | 23 | 20-40 | 124 | 76 | 74 | Secondary school | Single | Yes |
| D051 | Male | 42 | 40-60 | 115 | 69 | 53 | Secondary school | Married | Yes |
| D052 | Female | 23 | 20-40 | 120 | 75 | 49 | Higher education | Single | Yes |
| D053 | Female | 54 | 40-60 | 134 | 87 | 60 | Secondary school | Married | No |
| D054 | Female | 31 | 20-40 | 130 | 80 | 74 | Secondary school | Single | Yes |
| D055 | Male | 44 | 40-60 | 124 | 76 | 86 | Secondary school | Married | No |
| D056 | Male | 43 | 40-60 | 133 | 76 | 89 | Secondary school | Married | No |
| D057 | Male | 40 | 40-60 | 114 | 78 | 78 | Secondary school | Married | No |
| D058 | Male | 47 | 40-60 | 124 | 69 | 61 | Secondary school | Married | No |
| D059 | Male | 23 | 20-40 | 105 | 77 | 85 | Secondary school | Single | No |
| D060 | Male | 41 | 40-60 | 132 | 56 | 68 | Secondary school | Married | No |
| D061 | Male | 37 | 20-40 | 110 | 75 | 66 | Secondary school | Married | Yes |
| D062 | Female | 20 | 20-40 | 106 | 72 | 76 | Secondary school | Single | No |
| D063 | Female | 30 | 20-40 | 120 | 67 | 75 | Secondary school | Married | Yes |
| D064 | Female | 38 | 20-40 | 112 | 82 | 101 | Secondary school | Married | No |
| D065 | Male | 30 | 20-40 | 109 | 70 | 89 | Secondary school | Single | No |
| D066 | Female | 33 | 20-40 | 100 | 80 | 90 | Secondary school | Married | Yes |
| D067 | Female | 84 | ≥80 | 136 | 64 | 87 | Illiterate | Widow(er) | No |
| D068 | Female | 38 | 20-40 | 130 | 80 | 105 | Secondary school | Married | Yes |
| D069 | Male | 48 | 40-60 | 128 | 86 | 67 | Secondary school | Married | Yes |
| D070 | Female | 39 | 20-40 | 121 | 85 | 97 | Secondary school | Married | No |
| D071 | Female | 44 | 40-60 | 118 | 87 | 100 | Elementary school | Married | No |
| D072 | Female | 44 | 40-60 | 116 | 72 | 87 | Elementary school | Married | No |
| D074 | Female | 42 | 40-60 | 118 | 67 | 78 | Secondary school | Married | Yes |
| D075 | Female | 36 | 20-40 | 105 | 86 | 107 | Secondary school | Married | Yes |
| D076 | Male | 29 | 20-40 | 126 | 80 | 96 | Secondary school | Single | No |
| D077 | Female | 20 | 20-40 | 111 | 76 | 86 | Secondary school | Single | Yes |
| D078 | Male | 29 | 20-40 | 134 | 76 | 80 | Higher education | Single | No |
| D080 | Male | 81 | ≥80 | 130 | 90 | 77 | Elementary school | Widow(er) | No |
| D081 | Female | 29 | 20-40 | 127 | 77 | 87 | Secondary school | Married | No |
| D082 | Female | 51 | 40-60 | 128 | 89 | 88 | Secondary school | Married | Yes |
| D083 | Male | 35 | 20-40 | 128 | 77 | 102 | Secondary school | Single | No |
| D084 | Female | 51 | 40-60 | 134 | 87 | 84 | Secondary school | Married | Yes |
| D085 | Female | 42 | 40-60 | 117 | 72 | 99 | Secondary school | Married | No |
| D086 | Male | 64 | 60-80 | 138 | 87 | 64 | Secondary school | Widow(er) | No |
| D087 | Male | 56 | 40-60 | 127 | 80 | 76 | Secondary school | Married | Yes |
| D088 | Female | 66 | 60-80 | 122 | 64 | 51 | Secondary school | Married | Yes |
| D089 | Male | 34 | 20-40 | 118 | 83 | 64 | Secondary school | Married | No |
| D090 | Male | 58 | 40-60 | 130 | 66 | 80 | Elementary school | Married | Yes |
| D091 | Male | 54 | 40-60 | 120 | 70 | 73 | Higher education | Married | No |
| D093 | Male | 48 | 40-60 | 127 | 77 | 52 | Secondary school | Married | No |
| D094 | Male | 52 | 40-60 | 122 | 86 | 63 | Elementary school | Married | No |
| D095 | Female | 34 | 20-40 | 133 | 75 | 66 | Secondary school | Married | No |
| D096 | Female | 29 | 20-40 | 120 | 80 | 57 | Higher education | Single | Yes |
| D097 | Male | 25 | 20-40 | 112 | 68 | 67 | Secondary school | Single | No |
| D098 | Female | 55 | 40-60 | 125 | 75 | 86 | Secondary school | Married | Yes |
| D099 | Male | 41 | 40-60 | 132 | 79 | 92 | Elementary school | Married | No |
| D100 | Male | 48 | 40-60 | 130 | 68 | 46 | Secondary school | Married | No |
| D101 | Female | 42 | 40-60 | 123 | 63 | 90 | Secondary school | Married | No |
| D102 | Female | 20 | 20-40 | 110 | 66 | 89 | Secondary school | Single | No |
| D103 | Female | 23 | 20-40 | 108 | 70 | 79 | Secondary school | Single | No |
| D104 | Male | 47 | 40-60 | 112 | 69 | 80 | Secondary school | Married | Yes |
| D105 | Male | 20 | 20-40 | 102 | 70 | 67 | Secondary school | Single | Yes |
| D106 | Female | 68 | 60-80 | 128 | 77 | 107 | Elementary school | Widow(er) | No |
| D107 | Male | 39 | 20-40 | 110 | 65 | 100 | Secondary school | Married | No |
| D108 | Male | 47 | 40-60 | 117 | 60 | 68 | Secondary school | Married | Yes |
| D109 | Female | 36 | 20-40 | 100 | 76 | 95 | Secondary school | Married | No |
| D113 | Male | 32 | 20-40 | 104 | 66 | 97 | Secondary school | Single | No |
| D114 | Female | 80 | ≥80 | 115 | 79 | 99 | Illiterate | Widow(er) | No |
| D115 | Female | 53 | 40-60 | 107 | 82 | 87 | Secondary school | Married | No |
| D116 | Female | 29 | 20-40 | 102 | 82 | 104 | Secondary school | Married | Yes |
| D117 | Male | 66 | 60-80 | 115 | 78 | 89 | Elementary school | Married | No |
| D118 | Male | 71 | 60-80 | 128 | 87 | 70 | Illiterate | Widow(er) | No |
| D119 | Male | 42 | 40-60 | 130 | 78 | 85 | Secondary school | Married | Yes |
| D120 | Female | 28 | 20-40 | 120 | 78 | 98 | Secondary school | Married | Yes |
| D121 | Male | 33 | 20-40 | 111 | 80 | 86 | Secondary school | Married | Yes |
| D122 | Female | 72 | 60-80 | 121 | 74 | 80 | Illiterate | Widow(er) | No |
| D123 | Female | 60 | 60-80 | 123 | 73 | 93 | Elementary school | Widow(er) | No |
| D124 | Female | 70 | 60-80 | 110 | 70 | 87 | Elementary school | Widow(er) | No |
| D125 | Male | 29 | 20-40 | 105 | 86 | 90 | Secondary school | Single | Yes |
| D126 | Female | 35 | 20-40 | 127 | 79 | 98 | Secondary school | Married | Yes |
| D127 | Male | 46 | 40-60 | 133 | 67 | 107 | Secondary school | Married | No |
| D128 | Male | 39 | 20-40 | 109 | 67 | 110 | Secondary school | Married | No |
| D129 | Female | 65 | 60-80 | 102 | 80 | 85 | Elementary school | Widow(er) | No |
| D130 | Male | 44 | 40-60 | 120 | 65 | 65 | Secondary school | Married | No |
| D131 | Female | 65 | 60-80 | 102 | 78 | 60 | Illiterate | Widow(er) | No |
| D132 | Female | 44 | 40-60 | 119 | 76 | 80 | Secondary school | Married | No |
| D133 | Female | 66 | 60-80 | 105 | 66 | 65 | Elementary school | Widow(er) | No |
| D134 | Female | 54 | 40-60 | 100 | 80 | 76 | Secondary school | Married | Yes |
| D112 | Male | 35 | 20-40 | 112 | 77 | 89 | Secondary school | Married | No |
| D135 | Female | 33 | 20-40 | 110 | 70 | 78 | Secondary school | Married | Yes |
| Z001 | Male | 60 | 60-80 | 171 | 97 | 80 | Secondary school | Married | Yes |
| Z002 | Male | 81 | ≥80 | 156 | 95 | 87 | Elementary school | Married | Yes |
| z003 | Male | 32 | 20-40 | 144 | 99 | 102 | Secondary school | Married | Yes |
| Z004 | Female | 62 | 60-80 | 170 | 97 | 75 | Elementary school | Widow(er) | Yes |
| Z005 | Female | 66 | 60-80 | 111 | 88 | 121 | Elementary school | Married | No |
| Z006 | Female | 54 | 40-60 | 164 | 90 | 100 | Elementary school | Married | Yes |
| Z007 | Male | 53 | 40-60 | 158 | 78 | 79 | Secondary school | Married | Yes |
| Z008 | Male | 75 | 60-80 | 180 | 111 | 120 | Secondary school | Widow(er) | No |
| Z009 | Female | 55 | 40-60 | 158 | 98 | 99 | Secondary school | Married | Yes |
| Z010 | Male | 66 | 60-80 | 188 | 87 | 107 | Secondary school | Married | No |
| Z011 | Female | 60 | 60-80 | 149 | 84 | 84 | Secondary school | Married | No |
| Z012 | Female | 40 | 40-60 | 135 | 91 | 89 | Secondary school | Single | Yes |
| Z013 | Female | 74 | 60-80 | 231 | 101 | 110 | Elementary school | Widow(er) | No |
| Z014 | Female | 63 | 60-80 | 160 | 107 | 79 | Elementary school | Single | No |
| Z015 | Female | 40 | 40-60 | 188 | 100 | 87 | Secondary school | Single | Yes |
| Z016 | Male | 34 | 20-40 | 205 | 162 | 105 | Secondary school | Single | Yes |
| Z017 | Male | 50 | 40-60 | 144 | 90 | 70 | Secondary school | Married | No |
| Z018 | Female | 56 | 40-60 | 151 | 95 | 77 | Elementary school | Married | No |
| Z019 | Female | 67 | 60-80 | 180 | 121 | 103 | Illiterate | Widow(er) | Yes |
| Z020 | Female | 74 | 60-80 | 144 | 84 | 68 | Elementary school | Widow(er) | No |
| Z021 | Female | 43 | 40-60 | 164 | 87 | 101 | Secondary school | Married | Yes |
| Z022 | Female | 77 | 60-80 | 147 | 77 | 89 | Elementary school | Widow(er) | Yes |
| Z023 | Male | 53 | 40-60 | 145 | 105 | 100 | Secondary school | Married | Yes |
| Z024 | Female | 59 | 40-60 | 149 | 80 | 79 | Secondary school | Widow(er) | Yes |
| Z025 | Female | 66 | 60-80 | 185 | 92 | 87 | Secondary school | Widow(er) | Yes |
| Z026 | Female | 49 | 40-60 | 173 | 99 | 104 | Secondary school | Married | No |
| Z027 | Male | 66 | 60-80 | 153 | 94 | 87 | Elementary school | Married | Yes |
| Z028 | Female | 54 | 40-60 | 162 | 104 | 77 | Secondary school | Single | No |
| Z029 | Female | 39 | 20-40 | 149 | 94 | 80 | Secondary school | Married | Yes |
| Z030 | Female | 40 | 40-60 | 166 | 90 | 87 | Secondary school | Married | Yes |
| Z031 | Female | 58 | 40-60 | 224 | 118 | 98 | Secondary school | Widow(er) | No |
| Z032 | Female | 77 | 60-80 | 159 | 101 | 67 | Elementary school | Widow(er) | No |
| Z033 | Female | 54 | 40-60 | 152 | 85 | 85 | Elementary school | Married | Yes |
| Z034 | Female | 66 | 60-80 | 152 | 96 | 99 | Elementary school | Married | Yes |
| Z035 | Female | 65 | 60-80 | 204 | 107 | 110 | Elementary school | Widow(er) | Yes |
| Z036 | Male | 45 | 40-60 | 170 | 100 | 87 | Secondary school | Married | Yes |
| Z037 | Female | 60 | 60-80 | 149 | 84 | 88 | Elementary school | Widow(er) | Yes |
| Z038 | Male | 55 | 40-60 | 148 | 102 | 89 | Secondary school | Married | Yes |
| Z039 | Male | 47 | 40-60 | 173 | 114 | 90 | Secondary school | Married | Yes |
| Z040 | Female | 38 | 20-40 | 206 | 135 | 79 | Secondary school | Married | Yes |
| Z041 | Female | 64 | 60-80 | 154 | 62 | 60 | Elementary school | Married | Yes |
| Z042 | Female | 65 | 60-80 | 156 | 93 | 93 | Elementary school | Married | Yes |
| Z043 | Female | 62 | 60-80 | 194 | 89 | 109 | Illiterate | Married | Yes |
| Z044 | Female | 76 | 60-80 | 187 | 84 | 77 | Illiterate | Married | Yes |
| Z045 | Female | 63 | 60-80 | 229 | 147 | 78 | Elementary school | Married | Yes |
| Z046 | Male | 62 | 60-80 | 146 | 96 | 90 | Secondary school | Married | No |
| Z047 | Female | 54 | 40-60 | 269 | 156 | 72 | Secondary school | Married | No |
| Z048 | Male | 57 | 40-60 | 177 | 113 | 100 | Elementary school | Married | Yes |
| Z049 | Female | 52 | 40-60 | 254 | 141 | 102 | Secondary school | Married | Yes |
| Z050 | Female | 71 | 60-80 | 156 | 81 | 80 | Illiterate | Widow(er) | Yes |
| Z051 | Male | 48 | 40-60 | 143 | 101 | 80 | Secondary school | Married | Yes |
| Z052 | Female | 61 | 60-80 | 185 | 104 | 79 | Secondary school | Married | Yes |
| Z053 | Female | 43 | 40-60 | 208 | 131 | 102 | Elementary school | Widow(er) | Yes |
| Z054 | Female | 82 | ≥80 | 159 | 78 | 77 | Elementary school | Widow(er) | Yes |
| Z055 | Female | 63 | 60-80 | 167 | 91 | 90 | Secondary school | Widow(er) | Yes |
| Z056 | Female | 70 | 60-80 | 182 | 97 | 115 | Illiterate | Married | No |
| Z057 | Male | 51 | 40-60 | 165 | 103 | 77 | Secondary school | Married | No |
| Z058 | Female | 70 | 60-80 | 154 | 77 | 78 | Illiterate | Widow(er) | Yes |
| Z059 | Male | 64 | 60-80 | 146 | 90 | 115 | Secondary school | Married | Yes |
| Z060 | Male | 65 | 60-80 | 174 | 114 | 98 | Secondary school | Married | Yes |
| Z061 | Female | 55 | 40-60 | 154 | 94 | 66 | Secondary school | Married | Yes |
| Z062 | Male | 51 | 40-60 | 210 | 102 | 89 | Secondary school | Married | Yes |
| Z063 | Female | 59 | 40-60 | 168 | 100 | 80 | Illiterate | Married | Yes |
| Z064 | Female | 65 | 60-80 | 151 | 88 | 101 | Elementary school | Widow(er) | Yes |
| Z065 | Female | 70 | 60-80 | 176 | 87 | 99 | Secondary school | Married | Yes |
| Z066 | Female | 54 | 40-60 | 135 | 87 | 77 | Elementary school | Single | Yes |
| Z067 | Female | 68 | 60-80 | 160 | 87 | 102 | Elementary school | Widow(er) | Yes |
| Z068 | Female | 52 | 40-60 | 145 | 87 | 100 | Secondary school | Married | Yes |
| Z069 | Female | 87 | ≥80 | 180 | 102 | 87 | Secondary school | Widow(er) | Yes |
| Z070 | Female | 45 | 40-60 | 155 | 97 | 80 | Secondary school | Married | Yes |
| Z071 | Male | 49 | 40-60 | 167 | 77 | 102 | Secondary school | Married | Yes |
| Z072 | Female | 61 | 60-80 | 180 | 97 | 96 | Elementary school | Widow(er) | No |
| Z073 | Female | 55 | 40-60 | 142 | 81 | 100 | Illiterate | Widow(er) | Yes |
| Z074 | Female | 65 | 60-80 | 175 | 102 | 98 | Elementary school | Widow(er) | Yes |
| Z075 | Female | 65 | 60-80 | 144 | 95 | 100 | Elementary school | Widow(er) | No |
| Z076 | Female | 70 | 60-80 | 189 | 102 | 75 | Elementary school | Widow(er) | Yes |
| Z077 | Female | 56 | 40-60 | 179 | 102 | 98 | Secondary school | Married | No |
| Z078 | Female | 56 | 40-60 | 200 | 105 | 88 | Illiterate | Married | Yes |
| Z079 | Male | 74 | 60-80 | 146 | 97 | 77 | Elementary school | Single | Yes |
| Z080 | Female | 69 | 60-80 | 149 | 79 | 100 | Secondary school | Married | Yes |
| Z081 | Male | 37 | 20-40 | 141 | 95 | 89 | Higher education | Married | No |
| Z082 | Male | 47 | 40-60 | 141 | 96 | 100 | Secondary school | Married | Yes |
| Z083 | Female | 64 | 60-80 | 151 | 73 | 99 | Secondary school | Widow(er) | No |
| Z084 | Male | 60 | 60-80 | 207 | 108 | 110 | Secondary school | Married | Yes |
| Z085 | Female | 74 | 60-80 | 171 | 93 | 102 | Elementary school | Widow(er) | Yes |
| Z086 | Male | 69 | 60-80 | 184 | 99 | 110 | Secondary school | Married | Yes |
| Z087 | Female | 75 | 60-80 | 154 | 89 | 96 | Elementary school | Widow(er) | Yes |
| Z088 | Female | 41 | 40-60 | 141 | 93 | 98 | Secondary school | Married | Yes |
| Z089 | Female | 63 | 60-80 | 166 | 81 | 100 | Secondary school | Single | Yes |
| Z090 | Female | 37 | 20-40 | 166 | 105 | 100 | Elementary school | Married | No |
| Z091 | Female | 48 | 40-60 | 188 | 80 | 87 | Secondary school | Married | No |
| Z092 | Female | 61 | 60-80 | 164 | 104 | 78 | Secondary school | Married | No |
| Z093 | Male | 49 | 40-60 | 197 | 111 | 105 | Secondary school | Married | Yes |
| Z094 | Male | 68 | 60-80 | 162 | 102 | 78 | Elementary school | Married | Yes |
| Z096 | Male | 58 | 40-60 | 147 | 89 | 98 | Secondary school | Married | No |
| Te01 | Female | 65 | 60-80 | 117 | 71 | 78 | Elementary school | Widow(er) | No |
| Te02 | Female | 75 | 60-80 | 130 | 67 | 78 | Secondary school | Widow(er) | No |
| Te03 | Male | 38 | 20-40 | 122 | 75 | 102 | Higher education | Single | No |
| Te04 | Female | 32 | 20-40 | 116 | 74 | 68 | Secondary school | Single | Yes |
| Te05 | Male | 25 | 20-40 | 117 | 80 | 78 | Secondary school | Single | No |
| D150 | Female | 38 | 20-40 | 110 | 75 | 96 | Secondary school | Married | Yes |
| D151 | Female | 36 | 20-40 | 120 | 78 | 75 | Higher education | Married | No |
| D153 | Male | 28 | 20-40 | 115 | 89 | 100 | Secondary school | Married | No |
| D154 | Female | 22 | 20-40 | 123 | 74 | 80 | Secondary school | Single | No |
| D155 | Female | 38 | 20-40 | 129 | 84 | 87 | Elementary school | Single | No |
| D156 | Female | 20 | 20-40 | 130 | 77 | 76 | Secondary school | Single | Yes |
| D157 | Male | 35 | 20-40 | 123 | 87 | 90 | Secondary school | Single | Yes |
| D158 | Female | 40 | 40-60 | 105 | 100 | 78 | Higher education | Married | No |
| D159 | Female | 53 | 40-60 | 111 | 98 | 99 | Elementary school | Married | Yes |
| D160 | Female | 45 | 40-60 | 90 | 78 | 101 | Higher education | Married | No |
| D161 | Female | 36 | 20-40 | 114 | 85 | 95 | Higher education | Married | No |
| D162 | Male | 63 | 60-80 | 121 | 87 | 89 | Elementary school | Single | No |
| D163 | Female | 22 | 20-40 | 132 | 96 | 70 | Secondary school | Married | Yes |
| D164 | Female | 38 | 20-40 | 116 | 78 | 66 | Higher education | Single | Yes |
| D165 | Male | 57 | 40-60 | 102 | 84 | 77 | Secondary school | Married | Yes |
| D166 | Female | 20 | 20-40 | 102 | 97 | 90 | Higher education | Married | No |
| D167 | Female | 54 | 40-60 | 136 | 97 | 88 | Secondary school | Single | No |
| D168 | Female | 27 | 20-40 | 126 | 78 | 99 | Higher education | Married | Yes |
| D169 | Female | 25 | 20-40 | 101 | 88 | 88 | Secondary school | Single | No |
| D170 | Female | 58 | 40-60 | 107 | 79 | 76 | Secondary school | Married | No |
| D171 | Male | 27 | 20-40 | 120 | 69 | 99 | Higher education | Married | No |
| D172 | Female | 66 | 60-80 | 128 | 87 | 102 | Secondary school | Single | Yes |
| D174 | Female | 37 | 20-40 | 132 | 77 | 98 | Secondary school | Married | Yes |
| D175 | Female | 78 | 60-80 | 126 | 87 | 69 | Illiterate | Married | Yes |
| D176 | Male | 63 | 60-80 | 113 | 83 | 96 | Secondary school | Widow(er) | Yes |
| D177 | Male | 43 | 40-60 | 109 | 78 | 99 | Elementary school | Married | No |
| D178 | Female | 63 | 60-80 | 118 | 78 | 88 | Elementary school | Married | No |
| D179 | Male | 52 | 40-60 | 139 | 78 | 81 | Secondary school | Married | Yes |
| D180 | Female | 23 | 20-40 | 106 | 84 | 86 | Secondary school | Married | No |
| D181 | Male | 42 | 40-60 | 110 | 77 | 74 | Secondary school | Single | Yes |
| D182 | Female | 23 | 20-40 | 119 | 98 | 77 | Higher education | Married | No |
| D183 | Female | 54 | 40-60 | 109 | 87 | 88 | Secondary school | Single | Yes |
| D184 | Female | 31 | 20-40 | 103 | 84 | 67 | Secondary school | Married | No |
| D185 | Male | 44 | 40-60 | 98 | 65 | 77 | Secondary school | Single | Yes |
| D186 | Male | 43 | 40-60 | 104 | 88 | 87 | Secondary school | Married | No |
| D187 | Male | 40 | 40-60 | 112 | 86 | 64 | Secondary school | Married | No |
| D188 | Male | 47 | 40-60 | 105 | 74 | 107 | Higher education | Married | No |
| D190 | Male | 41 | 40-60 | 111 | 77 | 74 | Secondary school | Married | Yes |
| D191 | Male | 37 | 20-40 | 115 | 80 | 85 | Higher education | Married | No |
| D193 | Female | 30 | 20-40 | 125 | 87 | 96 | Secondary school | Married | Yes |
| D194 | Female | 38 | 20-40 | 130 | 74 | 86 | Higher education | Single | No |
| D195 | Male | 30 | 20-40 | 133 | 76 | 84 | Secondary school | Single | No |
| D196 | Female | 33 | 20-40 | 104 | 99 | 86 | Secondary school | Single | No |
| D197 | Female | 84 | ≥80 | 111 | 83 | 96 | Illiterate | Married | No |
| D198 | Female | 38 | 20-40 | 106 | 88 | 71 | Secondary school | Widow(er) | No |
| D199 | Male | 48 | 40-60 | 105 | 66 | 78 | Higher education | Single | No |
| D200 | Female | 39 | 20-40 | 127 | 70 | 75 | Elementary school | Married | Yes |
| Legend : code with T and Z, Hypertensive patients; code with D and Te, Normotensive patients **;** SBP : Systolic blood pressure ; DBP : Diastolic blood pressure; bpm: beats per minute. | | | | | | | | |  |

Material : research questionnaire form

Method : we used a questionnaire form to collect dada sex, ages, blood pressures, level of education, matrimonial status, and the family history of hypertension of the study participants.

**Suppl Table 2.** **Patients, hypertension status and treatments**

| Codes | Hypertension status | HTN grade | Therapy types | ACEI | Beta-blocker | CCB | ACEI+Diuretic | ACEI+CCB | Diuretic | ARB2 | ARB2+Diuretic | Calcium antagonist |
| --- | --- | --- | --- | --- | --- | --- | --- | --- | --- | --- | --- | --- |
| D201 | non-HTN | Non-HTN | Non-therapy | No | No | No | No | No | No | No | No | No |
| T002 | HTN | II | Monotherapy | No | No | Yes | No | No | No | No | No | No |
| T003 | HTN | III | Non-therapy | No | No | Yes | No | Yes | No | No | No | No |
| T004 | HTN | III | Monotherapy | No | No | Yes | No | No | Yes | No | No | No |
| D202 | non-HTN | Non-HTN | Non-therapy | No | No | No | No | No | No | No | No | No |
| T006 | HTN | I | Monotherapy | No | No | No | No | No | Yes | No | No | No |
| T007 | HTN | III | Monotherapy | No | Yes | No | No | No | No | No | No | No |
| D203 | non-HTN | Non-HTN | Non-therapy | No | No | No | No | No | No | No | No | No |
| D204 | non-HTN | Non-HTN | Non-therapy | No | No | No | No | No | No | No | No | No |
| D205 | non-HTN | Non-HTN | Non-therapy | No | No | No | No | No | No | No | No | No |
| T011 | HTN | III | Monotherapy | No | No | Yes | No | No | No | No | No | No |
| T012 | HTN | II | Bitherapy | No | Yes | No | No | No | No | No | No | No |
| T013 | HTN | II | Non-therapy | No | No | No | No | No | No | No | No | No |
| T014 | HTN | III | Monotherapy | Yes | No | Yes | No | No | No | No | No | No |
| T015 | HTN | III | Monotherapy | Yes | No | No | No | No | Yes | No | No | No |
| T016 | HTN | I | Monotherapy | No | No | No | No | No | Yes | No | No | No |
| T017 | HTN | III | Monotherapy | No | No | Yes | No | No | No | No | No | No |
| T018 | HTN | III | Monotherapy | No | No | No | No | No | Yes | Yes | No | No |
| T019 | HTN | III | Bitherapy | No | No | No | No | Yes | Yes | No | No | No |
| T020 | HTN | II | Non-therapy | No | No | No | No | No | No | No | No | No |
| T021 | HTN | II | Monotherapy | No | No | Yes | No | No | Yes | No | No | No |
| T022 | HTN | II | Bitherapy | No | No | Yes | Yes | No | No | No | No | No |
| T023 | HTN | III | Non-therapy | No | No | No | No | No | No | No | No | No |
| T024 | HTN | I | Monotherapy | No | Yes | Yes | No | No | No | No | No | No |
| T025 | HTN | I | Monotherapy | No | No | Yes | No | No | No | No | No | No |
| T026 | HTN | II | Bitherapy | No | No | No | Yes | Yes | No | No | No | No |
| T027 | HTN | II | Monotherapy | No | No | No | No | No | Yes | No | No | No |
| T028 | HTN | I | Bitherapy | No | No | No | No | Yes | No | No | No | No |
| D206 | non-HTN | Non-HTN | Non-therapy | No | No | No | No | No | No | No | No | No |
| T030 | HTN | III | Monotherapy | No | No | Yes | No | No | No | No | No | No |
| T031 | HTN | III | Bitherapy | No | No | No | Yes | No | No | No | No | No |
| T032 | HTN | II | Monotherapy | No | No | Yes | No | No | Yes | No | No | No |
| D207 | non-HTN | Non-HTN | Non-therapy | No | No | No | No | No | No | No | No | No |
| D208 | non-HTN | Non-HTN | Non-therapy | No | No | No | No | No | No | No | No | No |
| D209 | non-HTN | Non-HTN | Non-therapy | No | No | No | No | No | No | No | No | No |
| D210 | non-HTN | Non-HTN | Non-therapy | No | No | No | No | No | No | No | No | No |
| T037 | HTN | III | Monotherapy | No | No | No | No | No | Yes | No | No | No |
| T038 | HTN | III | Bitherapy | No | No | No | Yes | No | No | No | No | No |
| T039 | HTN | III | Non-therapy | No | No | No | No | No | No | No | No | No |
| T040 | HTN | I | Monotherapy | No | Yes | Yes | No | No | Yes | No | No | No |
| T041 | HTN | III | Monotherapy | No | No | Yes | No | No | No | No | No | No |
| T042 | HTN | III | Monotherapy | No | No | Yes | No | No | No | No | No | No |
| T043 | HTN | II | Monotherapy | No | No | Yes | No | No | No | No | No | No |
| T044 | HTN | II | Bitherapy | No | No | No | No | Yes | No | No | No | No |
| T045 | HTN | I | Monotherapy | Yes | No | Yes | No | No | No | No | No | No |
| T046 | HTN | I | Monotherapy | No | No | Yes | Yes | No | Yes | No | No | No |
| T047 | HTN | III | Non-therapy | No | No | No | No | No | No | No | No | No |
| T048 | HTN | I | Non-therapy | No | No | No | No | No | No | No | No | No |
| T049 | HTN | III | Monotherapy | No | Yes | Yes | No | No | Yes | No | No | No |
| T050 | HTN | III | Bitherapy | No | Yes | Yes | Yes | No | No | No | No | No |
| T051 | HTN | II | Monotherapy | No | No | Yes | No | No | No | No | No | No |
| T052 | HTN | I | Monotherapy | No | No | Yes | No | No | No | No | No | No |
| T053 | HTN | I | Monotherapy | No | Yes | Yes | No | No | No | No | No | No |
| T054 | HTN | I | Monotherapy | No | No | Yes | No | No | Yes | No | No | No |
| D211 | non-HTN | Non-HTN | Non-therapy | No | No | No | No | No | No | No | No | No |
| D212 | non-HTN | Non-HTN | Non-therapy | No | No | No | No | No | No | No | No | No |
| T057 | HTN | II | Monotherapy | Yes | No | Yes | No | No | Yes | No | No | No |
| T058 | HTN | III | Monotherapy | No | Yes | Yes | No | No | No | No | No | No |
| T059 | HTN | III | Monotherapy | No | No | Yes | Yes | No | No | No | No | No |
| T060 | HTN | I | Monotherapy | No | Yes | No | Yes | No | Yes | No | No | No |
| T061 | HTN | III | Monotherapy | No | Yes | Yes | Yes | No | No | No | No | No |
| T062 | HTN | III | Non-therapy | No | No | No | No | No | No | No | No | No |
| T063 | HTN | I | Bitherapy | No | No | No | No | No | No | No | No | No |
| T064 | HTN | III | Non-therapy | No | No | No | No | No | No | No | No | No |
| T065 | HTN | III | Monotherapy | No | Yes | Yes | No | No | Yes | No | No | No |
| T066 | HTN | I | Non-therapy | No | No | No | No | No | No | No | No | No |
| T067 | HTN | II | Monotherapy | No | No | Yes | No | No | No | No | No | No |
| T068 | HTN | III | Non-therapy | No | No | No | No | No | No | No | No | No |
| T069 | HTN | II | Monotherapy | No | No | Yes | No | No | Yes | No | No | No |
| T070 | HTN | I | Monotherapy | No | Yes | No | Yes | No | No | No | No | No |
| T071 | HTN | III | Monotherapy | No | No | Yes | No | No | No | No | No | No |
| T072 | HTN | I | Bitherapy | No | No | No | Yes | No | No | No | No | No |
| T073 | HTN | I | Non-therapy | No | No | No | No | No | No | No | No | No |
| T074 | HTN | III | Monotherapy | No | No | Yes | Yes | No | Yes | No | No | No |
| D213 | non-HTN | Non-HTN | Non-therapy | No | No | No | No | No | No | No | No | No |
| T076 | HTN | II | Bitherapy | No | No | Yes | No | No | No | No | No | No |
| T077 | HTN | II | Monotherapy | No | No | Yes | No | No | No | No | No | No |
| T078 | HTN | III | Monotherapy | No | No | No | No | No | No | No | No | Yes |
| T079 | HTN | III | Non-therapy | No | No | No | No | No | No | No | No | No |
| T080 | HTN | I | Monotherapy | No | No | Yes | Yes | No | Yes | No | No | No |
| T081 | HTN | III | Monotherapy | No | No | No | No | No | Yes | No | No | No |
| T082 | HTN | I | Non-therapy | No | Yes | No | No | No | Yes | No | No | No |
| T083 | HTN | III | Non-therapy | No | No | No | No | No | No | No | No | No |
| T084 | HTN | III | Monotherapy | No | No | Yes | No | No | No | No | No | No |
| T085 | HTN | III | Bitherapy | No | No | No | No | No | No | No | No | No |
| T086 | HTN | I | Non-therapy | No | No | No | No | No | No | No | No | No |
| T087 | HTN | I | Monotherapy | No | Yes | Yes | No | No | Yes | Yes | No | No |
| T088 | HTN | III | Non-therapy | No | No | No | No | No | No | No | No | No |
| T089 | HTN | III | Monotherapy | No | Yes | No | No | No | No | No | No | No |
| T090 | HTN | III | Non-therapy | No | No | No | No | No | No | No | No | No |
| T091 | HTN | III | Non-therapy | No | No | No | No | No | No | No | No | No |
| T092 | HTN | III | Non-therapy | No | No | No | No | No | No | No | No | No |
| D214 | non-HTN | Non-HTN | Non-therapy | No | No | No | No | No | No | No | No | No |
| T094 | HTN | I | Monotherapy | No | No | Yes | No | No | No | No | No | No |
| T095 | HTN | III | Monotherapy | No | No | Yes | No | No | No | No | No | No |
| T096 | HTN | I | Monotherapy | No | No | Yes | No | No | No | No | No | No |
| T097 | HTN | II | Monotherapy | Yes | No | No | No | No | Yes | No | No | No |
| T098 | HTN | I | Monotherapy | No | No | Yes | No | No | No | No | No | No |
| T099 | HTN | II | Monotherapy | No | No | Yes | Yes | No | No | No | No | No |
| T100 | HTN | II | Bitherapy | No | No | No | Yes | No | No | No | No | No |
| T101 | HTN | III | Monotherapy | No | Yes | No | No | No | No | No | No | No |
| T102 | HTN | I | Tritherapy | No | No | No | No | No | No | No | No | No |
| T103 | HTN | II | Tritherapy | No | No | No | No | No | No | No | No | No |
| T104 | HTN | III | Tritherapy | No | No | No | No | No | No | No | No | No |
| T105 | HTN | III | Monotherapy | No | No | Yes | No | No | Yes | No | No | No |
| T106 | HTN | II | Bitherapy | No | No | No | No | No | No | No | No | No |
| T107 | HTN | III | Non-therapy | No | No | No | No | No | No | No | No | No |
| T108 | HTN | II | Tritherapy | No | No | No | No | No | No | No | No | No |
| T109 | HTN | II | Monotherapy | No | Yes | No | No | No | No | No | No | No |
| T110 | HTN | III | Non-therapy | No | No | No | No | No | No | No | No | No |
| T111 | HTN | III | Non-therapy | No | No | No | No | No | No | No | No | No |
| T112 | HTN | III | Non-therapy | No | No | No | No | No | No | No | No | No |
| T113 | HTN | III | Non-therapy | No | No | No | No | No | No | No | No | No |
| T114 | HTN | I | Bitherapy | No | No | No | Yes | No | No | No | No | No |
| T115 | HTN | III | Bitherapy | No | No | No | No | No | No | No | No | No |
| T116 | HTN | III | Tritherapy | No | No | Yes | No | No | No | No | No | No |
| T117 | HTN | III | Non-therapy | No | No | No | No | No | No | No | No | No |
| T118 | HTN | III | Monotherapy | No | Yes | No | No | No | Yes | No | No | No |
| T119 | HTN | II | Monotherapy | No | Yes | No | No | No | No | No | No | No |
| T120 | HTN | III | Monotherapy | No | No | Yes | No | No | No | No | No | No |
| T121 | HTN | I | Bitherapy | No | No | No | No | No | No | No | Yes | No |
| T122 | HTN | III | Non-therapy | No | No | No | No | No | No | No | No | No |
| T123 | HTN | III | Non-therapy | No | No | No | No | No | No | No | No | No |
| T124 | HTN | III | Monotherapy | No | No | Yes | No | No | No | No | No | No |
| T125 | HTN | III | Monotherapy | No | No | Yes | No | No | No | No | No | No |
| T126 | HTN | III | Monotherapy | No | Yes | Yes | No | Yes | No | No | No | No |
| T127 | HTN | III | Bitherapy | No | No | No | Yes | No | No | No | No | No |
| T128 | HTN | III | Non-therapy | No | No | No | No | No | No | No | No | No |
| T129 | HTN | III | Non-therapy | No | No | No | No | No | No | No | No | No |
| T130 | HTN | I | Monotherapy | No | No | Yes | No | No | No | No | No | No |
| T131 | HTN | III | Monotherapy | No | Yes | No | No | No | No | No | No | No |
| T132 | HTN | II | Non-therapy | No | No | No | No | No | No | No | No | No |
| T133 | HTN | III | Bitherapy | No | No | No | Yes | No | No | No | No | No |
| T134 | HTN | III | Bitherapy | No | No | No | Yes | No | No | No | No | No |
| T135 | HTN | III | Monotherapy | No | No | Yes | No | No | No | No | No | No |
| T136 | HTN | II | Monotherapy | No | No | No | No | No | Yes | No | No | No |
| T137 | HTN | I | Monotherapy | No | No | Yes | No | No | No | No | No | No |
| T138 | HTN | II | Non-therapy | No | No | No | No | No | No | No | No | No |
| T139 | HTN | I | Non-therapy | No | No | No | No | No | No | No | No | No |
| T140 | HTN | I | Monotherapy | No | Yes | No | No | No | No | No | No | No |
| T141 | HTN | III | Bitherapy | No | No | No | Yes | No | No | No | No | No |
| T142 | HTN | II | Monotherapy | No | No | Yes | No | No | No | No | No | No |
| T143 | HTN | I | Non-therapy | No | No | No | No | No | No | No | No | No |
| T144 | HTN | II | Bitherapy | No | No | No | Yes | No | No | No | No | No |
| T145 | HTN | I | Non-therapy | No | No | No | No | No | No | No | No | No |
| T146 | HTN | I | Non-therapy | No | No | No | No | No | No | No | No | No |
| T147 | HTN | II | Monotherapy | No | No | Yes | No | No | No | No | No | No |
| T148 | HTN | II | Monotherapy | No | No | No | No | No | No | No | No | Yes |
| T149 | HTN | III | Monotherapy | No | No | Yes | No | No | No | No | No | No |
| T150 | HTN | I | Monotherapy | No | No | Yes | No | No | No | No | No | No |
| T151 | HTN | II | Bitherapy | No | Yes | No | No | No | No | No | No | No |
| T152 | HTN | I | Monotherapy | No | No | Yes | No | No | No | No | No | No |
| T153 | HTN | I | Monotherapy | No | No | Yes | No | No | No | No | No | No |
| T154 | HTN | III | Monotherapy | No | No | Yes | No | No | No | No | No | No |
| T155 | HTN | II | Bitherapy | No | No | No | Yes | No | No | No | No | No |
| T156 | HTN | III | Monotherapy | No | No | Yes | No | No | No | No | No | No |
| T157 | HTN | III | Non-therapy | No | No | No | No | No | No | No | No | No |
| T158 | HTN | II | Bitherapy | No | Yes | No | Yes | No | No | No | No | No |
| T159 | HTN | I | Monotherapy | Yes | No | No | No | No | No | No | No | No |
| T160 | HTN | III | Monotherapy | No | Yes | Yes | No | No | No | No | No | No |
| T161 | HTN | III | Monotherapy | No | Yes | No | No | No | No | No | No | No |
| T162 | HTN | III | Monotherapy | No | No | Yes | No | No | No | No | No | No |
| T163 | HTN | II | Bitherapy | No | No | No | No | Yes | No | No | No | No |
| T164 | HTN | II | Non-therapy | No | No | No | No | No | No | No | No | No |
| T165 | HTN | III | Non-therapy | No | No | No | No | No | No | No | No | No |
| T166 | HTN | I | Monotherapy | No | No | Yes | No | No | No | No | No | No |
| T167 | HTN | II | Monotherapy | No | No | No | No | No | No | Yes | No | No |
| T168 | HTN | I | Monotherapy | No | No | Yes | No | No | No | No | No | No |
| T169 | HTN | II | Non-therapy | No | No | No | No | No | No | No | No | No |
| T170 | HTN | I | Monotherapy | No | No | Yes | Yes | No | No | No | No | No |
| T171 | HTN | I | Monotherapy | No | No | Yes | No | No | Yes | No | No | No |
| T172 | HTN | I | Non-therapy | No | No | No | No | No | No | No | No | No |
| T173 | HTN | III | Monotherapy | No | Yes | No | No | No | No | No | No | No |
| T174 | HTN | I | Non-therapy | No | No | No | No | No | No | No | No | No |
| T175 | HTN | II | Bitherapy | No | No | No | No | Yes | No | No | No | No |
| T176 | HTN | III | Non-therapy | No | No | No | No | No | No | No | No | No |
| T177 | HTN | II | Non-therapy | No | No | No | No | No | No | No | No | No |
| T178 | HTN | III | Bitherapy | No | No | No | No | No | No | No | No | No |
| T179 | HTN | I | Monotherapy | No | Yes | Yes | No | No | Yes | No | No | No |
| T180 | HTN | I | Bitherapy | No | No | Yes | No | No | No | No | Yes | No |
| T181 | HTN | III | Monotherapy | No | No | Yes | No | No | No | No | No | No |
| T182 | HTN | I | Monotherapy | No | No | Yes | No | No | No | No | No | No |
| T183 | HTN | I | Monotherapy | No | No | No | No | No | No | No | No | No |
| T184 | HTN | III | Bitherapy | No | No | No | No | No | No | No | No | No |
| T185 | HTN | III | Tritherapy | No | No | No | No | No | No | No | No | No |
| T186 | HTN | II | Monotherapy | No | No | No | No | No | No | No | No | No |
| T187 | HTN | III | Monotherapy | No | No | Yes | No | No | No | No | No | No |
| T188 | HTN | I | Bitherapy | No | No | No | No | No | No | No | No | No |
| T189 | HTN | III | Monotherapy | Yes | No | No | No | No | No | No | No | No |
| T190 | HTN | I | Non-therapy | No | No | No | No | No | No | No | No | No |
| T191 | HTN | II | Non-therapy | No | No | No | No | No | No | No | No | No |
| T192 | HTN | II | Bitherapy | No | No | No | No | No | No | No | No | No |
| T193 | HTN | III | Monotherapy | No | No | Yes | No | No | No | No | No | No |
| T194 | HTN | I | Non-therapy | No | No | No | No | No | No | No | No | No |
| T195 | HTN | I | Non-therapy | No | No | No | No | No | No | No | No | No |
| T196 | HTN | I | Non-therapy | No | No | No | No | No | No | No | No | No |
| T197 | HTN | I | Bitherapy | No | No | No | Yes | No | No | No | No | No |
| T198 | HTN | III | Non-therapy | No | No | No | No | No | No | No | No | No |
| T199 | HTN | I | Non-therapy | No | No | No | No | No | No | No | No | No |
| T200 | HTN | II | Monotherapy | No | No | Yes | No | No | No | No | No | No |
| T201 | HTN | I | Monotherapy | No | No | No | No | No | Yes | No | No | No |
| T202 | HTN | II | Monotherapy | No | No | Yes | No | No | No | No | No | No |
| T203 | HTN | I | Monotherapy | Yes | Yes | No | No | No | No | No | No | No |
| T204 | HTN | III | Non-therapy | No | No | No | No | No | No | No | No | No |
| T205 | HTN | III | Monotherapy | No | No | Yes | No | No | No | No | No | No |
| T206 | HTN | I | Bitherapy | No | No | No | No | No | No | No | No | No |
| T207 | HTN | II | Monotherapy | No | No | Yes | No | No | No | No | No | No |
| T208 | HTN | III | Monotherapy | No | No | Yes | No | No | No | No | No | No |
| T209 | HTN | I | Monotherapy | No | Yes | No | No | No | No | No | No | No |
| T210 | HTN | I | Monotherapy | No | Yes | No | No | No | No | No | No | No |
| T211 | HTN | I | Monotherapy | No | No | No | No | No | No | No | No | No |
| T212 | HTN | I | Monotherapy | No | No | Yes | No | No | No | No | No | No |
| T213 | HTN | I | Non-therapy | No | No | No | No | No | No | No | No | No |
| T214 | HTN | II | Monotherapy | No | Yes | No | No | No | No | No | No | No |
| T215 | HTN | III | Non-therapy | No | No | No | No | No | No | No | No | No |
| T216 | HTN | I | Non-therapy | No | No | No | No | No | No | No | No | No |
| T217 | HTN | II | Non-therapy | No | No | No | No | No | No | No | No | No |
| T218 | HTN | I | Monotherapy | No | Yes | No | No | No | No | No | No | No |
| T219 | HTN | I | Non-therapy | No | No | No | No | No | No | No | No | No |
| T220 | HTN | II | Bitherapy | No | No | No | No | No | No | No | No | No |
| T221 | HTN | I | Bitherapy | No | No | No | Yes | No | No | No | No | No |
| T222 | HTN | I | Bitherapy | No | No | No | Yes | No | No | No | No | No |
| T223 | HTN | III | Non-therapy | No | No | No | No | No | No | No | No | No |
| T224 | HTN | II | Monotherapy | No | No | Yes | No | No | No | No | No | No |
| T225 | HTN | III | Non-therapy | No | No | No | No | No | No | No | No | No |
| T226 | HTN | II | Monotherapy | No | No | Yes | No | No | No | No | No | No |
| T227 | HTN | III | Bitherapy | No | No | Yes | Yes | No | No | No | No | No |
| T228 | HTN | I | Monotherapy | No | No | Yes | No | No | No | No | No | No |
| T229 | HTN | I | Monotherapy | No | Yes | Yes | No | No | No | No | No | No |
| T230 | HTN | III | Bitherapy | No | No | No | No | No | No | No | No | No |
| T231 | HTN | I | Monotherapy | No | No | No | No | No | Yes | No | No | No |
| T232 | HTN | I | Non-therapy | No | No | No | No | No | No | No | No | No |
| T233 | HTN | I | Non-therapy | No | No | No | No | No | No | No | No | No |
| T234 | HTN | III | Bitherapy | No | No | No | Yes | No | No | No | No | No |
| T235 | HTN | III | Non-therapy | No | No | No | No | No | No | No | No | No |
| T236 | HTN | II | Non-therapy | No | No | No | No | No | No | No | No | No |
| T237 | HTN | II | Monotherapy | No | No | Yes | No | No | No | No | No | No |
| T238 | HTN | II | Non-therapy | No | No | No | No | No | No | No | No | No |
| T239 | HTN | II | Monotherapy | No | Yes | No | No | No | No | No | No | No |
| T240 | HTN | II | Non-therapy | No | No | No | No | No | No | No | No | No |
| T241 | HTN | III | Monotherapy | No | No | Yes | No | No | No | No | No | No |
| T242 | HTN | III | Monotherapy | No | Yes | No | No | No | No | No | No | No |
| T243 | HTN | III | Non-therapy | No | No | No | No | No | No | No | No | No |
| T244 | HTN | I | Non-therapy | No | No | No | No | No | No | No | No | No |
| T245 | HTN | I | Monotherapy | No | No | No | No | No | No | No | No | No |
| D001 | non-HTN | Non-HTN | Non-therapy | No | No | No | No | No | No | No | No | No |
| D002 | non-HTN | Non-HTN | Non-therapy | No | No | No | No | No | No | No | No | No |
| D003 | non-HTN | Non-HTN | Non-therapy | No | No | No | No | No | No | No | No | No |
| D004 | non-HTN | Non-HTN | Non-therapy | No | No | No | No | No | No | No | No | No |
| D005 | non-HTN | Non-HTN | Non-therapy | No | No | No | No | No | No | No | No | No |
| D006 | non-HTN | Non-HTN | Non-therapy | No | No | No | No | No | No | No | No | No |
| D007 | non-HTN | Non-HTN | Non-therapy | No | No | No | No | No | No | No | No | No |
| D008 | non-HTN | Non-HTN | Non-therapy | No | No | No | No | No | No | No | No | No |
| D009 | non-HTN | Non-HTN | Non-therapy | No | No | No | No | No | No | No | No | No |
| D011 | non-HTN | Non-HTN | Non-therapy | No | No | No | No | No | No | No | No | No |
| D012 | non-HTN | Non-HTN | Non-therapy | No | No | No | No | No | No | No | No | No |
| D013 | non-HTN | Non-HTN | Non-therapy | No | No | No | No | No | No | No | No | No |
| D014 | non-HTN | Non-HTN | Non-therapy | No | No | No | No | No | No | No | No | No |
| D015 | non-HTN | Non-HTN | Non-therapy | No | No | No | No | No | No | No | No | No |
| D016 | non-HTN | Non-HTN | Non-therapy | No | No | No | No | No | No | No | No | No |
| D017 | non-HTN | Non-HTN | Non-therapy | No | No | No | No | No | No | No | No | No |
| D018 | non-HTN | Non-HTN | Non-therapy | No | No | No | No | No | No | No | No | No |
| D019 | non-HTN | Non-HTN | Non-therapy | No | No | No | No | No | No | No | No | No |
| D020 | non-HTN | Non-HTN | Non-therapy | No | No | No | No | No | No | No | No | No |
| D021 | non-HTN | Non-HTN | Non-therapy | No | No | No | No | No | No | No | No | No |
| D022 | non-HTN | Non-HTN | Non-therapy | No | No | No | No | No | No | No | No | No |
| D023 | non-HTN | Non-HTN | Non-therapy | No | No | No | No | No | No | No | No | No |
| D024 | non-HTN | Non-HTN | Non-therapy | No | No | No | No | No | No | No | No | No |
| D025 | non-HTN | Non-HTN | Non-therapy | No | No | No | No | No | No | No | No | No |
| D027 | non-HTN | Non-HTN | Non-therapy | No | No | No | No | No | No | No | No | No |
| D028 | non-HTN | Non-HTN | Non-therapy | No | No | No | No | No | No | No | No | No |
| D029 | non-HTN | Non-HTN | Non-therapy | No | No | No | No | No | No | No | No | No |
| D030 | non-HTN | Non-HTN | Non-therapy | No | No | No | No | No | No | No | No | No |
| D031 | non-HTN | Non-HTN | Non-therapy | No | No | No | No | No | No | No | No | No |
| T246 | HTN | I | Monotherapy | No | No | Yes | No | No | No | No | No | No |
| D034 | non-HTN | Non-HTN | Non-therapy | No | No | No | No | No | No | No | No | No |
| D035 | non-HTN | Non-HTN | Non-therapy | No | No | No | No | No | No | No | No | No |
| D036 | non-HTN | Non-HTN | Non-therapy | No | No | No | No | No | No | No | No | No |
| D037 | non-HTN | Non-HTN | Non-therapy | No | No | No | No | No | No | No | No | No |
| D038 | non-HTN | Non-HTN | Non-therapy | No | No | No | No | No | No | No | No | No |
| D039 | non-HTN | Non-HTN | Non-therapy | No | No | No | No | No | No | No | No | No |
| D040 | non-HTN | Non-HTN | Non-therapy | No | No | No | No | No | No | No | No | No |
| D041 | non-HTN | Non-HTN | Non-therapy | No | No | No | No | No | No | No | No | No |
| D042 | non-HTN | Non-HTN | Non-therapy | No | No | No | No | No | No | No | No | No |
| D044 | non-HTN | Non-HTN | Non-therapy | No | No | No | No | No | No | No | No | No |
| D045 | non-HTN | Non-HTN | Non-therapy | No | No | No | No | No | No | No | No | No |
| D046 | non-HTN | Non-HTN | Non-therapy | No | No | No | No | No | No | No | No | No |
| D047 | non-HTN | Non-HTN | Non-therapy | No | No | No | No | No | No | No | No | No |
| D048 | non-HTN | Non-HTN | Non-therapy | No | No | No | No | No | No | No | No | No |
| D049 | non-HTN | Non-HTN | Non-therapy | No | No | No | No | No | No | No | No | No |
| D050 | non-HTN | Non-HTN | Non-therapy | No | No | No | No | No | No | No | No | No |
| D051 | non-HTN | Non-HTN | Non-therapy | No | No | No | No | No | No | No | No | No |
| D052 | non-HTN | Non-HTN | Non-therapy | No | No | No | No | No | No | No | No | No |
| D053 | non-HTN | Non-HTN | Non-therapy | No | No | No | No | No | No | No | No | No |
| D054 | non-HTN | Non-HTN | Non-therapy | No | No | No | No | No | No | No | No | No |
| D055 | non-HTN | Non-HTN | Non-therapy | No | No | No | No | No | No | No | No | No |
| D056 | non-HTN | Non-HTN | Non-therapy | No | No | No | No | No | No | No | No | No |
| D057 | non-HTN | Non-HTN | Non-therapy | No | No | No | No | No | No | No | No | No |
| D058 | non-HTN | Non-HTN | Non-therapy | No | No | No | No | No | No | No | No | No |
| D059 | non-HTN | Non-HTN | Non-therapy | No | No | No | No | No | No | No | No | No |
| D060 | non-HTN | Non-HTN | Non-therapy | No | No | No | No | No | No | No | No | No |
| D061 | non-HTN | Non-HTN | Non-therapy | No | No | No | No | No | No | No | No | No |
| D062 | non-HTN | Non-HTN | Non-therapy | No | No | No | No | No | No | No | No | No |
| D063 | non-HTN | Non-HTN | Non-therapy | No | No | No | No | No | No | No | No | No |
| D064 | non-HTN | Non-HTN | Non-therapy | No | No | No | No | No | No | No | No | No |
| D065 | non-HTN | Non-HTN | Non-therapy | No | No | No | No | No | No | No | No | No |
| D066 | non-HTN | Non-HTN | Non-therapy | No | No | No | No | No | No | No | No | No |
| D067 | non-HTN | Non-HTN | Non-therapy | No | No | No | No | No | No | No | No | No |
| D068 | non-HTN | Non-HTN | Non-therapy | No | No | No | No | No | No | No | No | No |
| D069 | non-HTN | Non-HTN | Non-therapy | No | No | No | No | No | No | No | No | No |
| D070 | non-HTN | Non-HTN | Non-therapy | No | No | No | No | No | No | No | No | No |
| D071 | non-HTN | Non-HTN | Non-therapy | No | No | No | No | No | No | No | No | No |
| D072 | non-HTN | Non-HTN | Non-therapy | No | No | No | No | No | No | No | No | No |
| D074 | non-HTN | Non-HTN | Non-therapy | No | No | No | No | No | No | No | No | No |
| D075 | non-HTN | Non-HTN | Non-therapy | No | No | No | No | No | No | No | No | No |
| D076 | non-HTN | Non-HTN | Non-therapy | No | No | No | No | No | No | No | No | No |
| D077 | non-HTN | Non-HTN | Non-therapy | No | No | No | No | No | No | No | No | No |
| D078 | non-HTN | Non-HTN | Non-therapy | No | No | No | No | No | No | No | No | No |
| D080 | non-HTN | Non-HTN | Non-therapy | No | No | No | No | No | No | No | No | No |
| D081 | non-HTN | Non-HTN | Non-therapy | No | No | No | No | No | No | No | No | No |
| D082 | non-HTN | Non-HTN | Non-therapy | No | No | No | No | No | No | No | No | No |
| D083 | non-HTN | Non-HTN | Non-therapy | No | No | No | No | No | No | No | No | No |
| D084 | non-HTN | Non-HTN | Non-therapy | No | No | No | No | No | No | No | No | No |
| D085 | non-HTN | Non-HTN | Non-therapy | No | No | No | No | No | No | No | No | No |
| D086 | non-HTN | Non-HTN | Non-therapy | No | No | No | No | No | No | No | No | No |
| D087 | non-HTN | Non-HTN | Non-therapy | No | No | No | No | No | No | No | No | No |
| D088 | non-HTN | Non-HTN | Non-therapy | No | No | No | No | No | No | No | No | No |
| D089 | non-HTN | Non-HTN | Non-therapy | No | No | No | No | No | No | No | No | No |
| D090 | non-HTN | Non-HTN | Non-therapy | No | No | No | No | No | No | No | No | No |
| D091 | non-HTN | Non-HTN | Non-therapy | No | No | No | No | No | No | No | No | No |
| D093 | non-HTN | Non-HTN | Non-therapy | No | No | No | No | No | No | No | No | No |
| D094 | non-HTN | Non-HTN | Non-therapy | No | No | No | No | No | No | No | No | No |
| D095 | non-HTN | Non-HTN | Non-therapy | No | No | No | No | No | No | No | No | No |
| D096 | non-HTN | Non-HTN | Non-therapy | No | No | No | No | No | No | No | No | No |
| D097 | non-HTN | Non-HTN | Non-therapy | No | No | No | No | No | No | No | No | No |
| D098 | non-HTN | Non-HTN | Non-therapy | No | No | No | No | No | No | No | No | No |
| D099 | non-HTN | Non-HTN | Non-therapy | No | No | No | No | No | No | No | No | No |
| D100 | non-HTN | Non-HTN | Non-therapy | No | No | No | No | No | No | No | No | No |
| D101 | non-HTN | Non-HTN | Non-therapy | No | No | No | No | No | No | No | No | No |
| D102 | non-HTN | Non-HTN | Non-therapy | No | No | No | No | No | No | No | No | No |
| D103 | non-HTN | Non-HTN | Non-therapy | No | No | No | No | No | No | No | No | No |
| D104 | non-HTN | Non-HTN | Non-therapy | No | No | No | No | No | No | No | No | No |
| D105 | non-HTN | Non-HTN | Non-therapy | No | No | No | No | No | No | No | No | No |
| D106 | non-HTN | Non-HTN | Non-therapy | No | No | No | No | No | No | No | No | No |
| D107 | non-HTN | Non-HTN | Non-therapy | No | No | No | No | No | No | No | No | No |
| D108 | non-HTN | Non-HTN | Non-therapy | No | No | No | No | No | No | No | No | No |
| D109 | non-HTN | Non-HTN | Non-therapy | No | No | No | No | No | No | No | No | No |
| D113 | non-HTN | Non-HTN | Non-therapy | No | No | No | No | No | No | No | No | No |
| D114 | non-HTN | Non-HTN | Non-therapy | No | No | No | No | No | No | No | No | No |
| D115 | non-HTN | Non-HTN | Non-therapy | No | No | No | No | No | No | No | No | No |
| D116 | non-HTN | Non-HTN | Non-therapy | No | No | No | No | No | No | No | No | No |
| D117 | non-HTN | Non-HTN | Non-therapy | No | No | No | No | No | No | No | No | No |
| D118 | non-HTN | Non-HTN | Non-therapy | No | No | No | No | No | No | No | No | No |
| D119 | non-HTN | Non-HTN | Non-therapy | No | No | No | No | No | No | No | No | No |
| D120 | non-HTN | Non-HTN | Non-therapy | No | No | No | No | No | No | No | No | No |
| D121 | non-HTN | Non-HTN | Non-therapy | No | No | No | No | No | No | No | No | No |
| D122 | non-HTN | Non-HTN | Non-therapy | No | No | No | No | No | No | No | No | No |
| D123 | non-HTN | Non-HTN | Non-therapy | No | No | No | No | No | No | No | No | No |
| D124 | non-HTN | Non-HTN | Non-therapy | No | No | No | No | No | No | No | No | No |
| D125 | non-HTN | Non-HTN | Non-therapy | No | No | No | No | No | No | No | No | No |
| D126 | non-HTN | Non-HTN | Non-therapy | No | No | No | No | No | No | No | No | No |
| D127 | non-HTN | Non-HTN | Non-therapy | No | No | No | No | No | No | No | No | No |
| D128 | non-HTN | Non-HTN | Non-therapy | No | No | No | No | No | No | No | No | No |
| D129 | non-HTN | Non-HTN | Non-therapy | No | No | No | No | No | No | No | No | No |
| D130 | non-HTN | Non-HTN | Non-therapy | No | No | No | No | No | No | No | No | No |
| D131 | non-HTN | Non-HTN | Non-therapy | No | No | No | No | No | No | No | No | No |
| D132 | non-HTN | Non-HTN | Non-therapy | No | No | No | No | No | No | No | No | No |
| D133 | non-HTN | Non-HTN | Non-therapy | No | No | No | No | No | No | No | No | No |
| D134 | non-HTN | Non-HTN | Non-therapy | No | No | No | No | No | No | No | No | No |
| D112 | non-HTN | Non-HTN | Non-therapy | No | No | No | No | No | No | No | No | No |
| D135 | non-HTN | Non-HTN | Non-therapy | No | No | No | No | No | No | No | No | No |
| Z001 | HTN | II | Monotherapy | No | Yes | Yes | No | No | No | No | No | No |
| Z002 | HTN | I | Monotherapy | No | No | Yes | No | No | No | No | No | No |
| z003 | HTN | I | Monotherapy | No | No | Yes | No | No | No | No | No | No |
| Z004 | HTN | II | Monotherapy | No | No | Yes | No | No | No | No | No | No |
| Z005 | HTN | I | Monotherapy | No | No | Yes | No | No | No | No | No | No |
| Z006 | HTN | II | Non-therapy | No | No | No | No | No | No | No | No | No |
| Z007 | HTN | I | Non-therapy | No | No | No | No | No | No | No | No | No |
| Z008 | HTN | III | Bitherapy | No | No | No | Yes | No | No | No | No | No |
| Z009 | HTN | I | Monotherapy | No | No | Yes | No | No | No | No | No | No |
| Z010 | HTN | II | Monotherapy | No | No | Yes | No | No | No | No | No | No |
| Z011 | HTN | I | Bitherapy | No | No | No | Yes | No | No | No | No | No |
| Z012 | HTN | I | Monotherapy | No | No | Yes | No | No | No | No | No | No |
| Z013 | HTN | III | Bitherapy | No | No | No | Yes | No | No | No | No | No |
| Z014 | HTN | II | Monotherapy | No | No | Yes | No | No | No | No | No | No |
| Z015 | HTN | III | Non-therapy | No | No | No | No | No | No | No | No | No |
| Z016 | HTN | III | Non-therapy | No | No | No | No | No | No | No | No | No |
| Z017 | HTN | I | Tritherapy | No | No | No | No | No | No | No | No | No |
| Z018 | HTN | I | Monotherapy | No | No | Yes | No | No | No | No | No | No |
| Z019 | HTN | III | Bitherapy | No | No | No | Yes | No | No | No | No | No |
| Z020 | HTN | I | Monotherapy | No | No | Yes | No | No | No | No | No | No |
| Z021 | HTN | II | Monotherapy | No | No | Yes | No | No | No | No | No | No |
| Z022 | HTN | I | Bitherapy | No | No | No | No | Yes | No | No | No | No |
| Z023 | HTN | II | Monotherapy | No | No | No | No | No | No | No | No | No |
| Z024 | HTN | I | Bitherapy | No | No | No | No | No | No | No | No | No |
| Z025 | HTN | III | Non-therapy | No | No | No | No | No | No | No | No | No |
| Z026 | HTN | II | Monotherapy | No | Yes | No | No | No | No | No | No | No |
| Z027 | HTN | II | Non-therapy | No | No | No | No | No | No | No | No | No |
| Z028 | HTN | II | Non-therapy | No | No | No | No | No | No | No | No | No |
| Z029 | HTN | I | Monotherapy | Yes | No | Yes | No | No | No | No | No | No |
| Z030 | HTN | II | Bitherapy | No | No | No | No | No | No | No | No | No |
| Z031 | HTN | III | Bitherapy | No | No | Yes | No | No | Yes | No | No | No |
| Z032 | HTN | I | Bitherapy | No | No | No | Yes | No | No | No | No | No |
| Z033 | HTN | I | Non-therapy | No | No | No | No | No | No | No | No | No |
| Z034 | HTN | I | Monotherapy | Yes | No | No | No | No | No | No | No | No |
| Z035 | HTN | III | Tritherapy | No | No | No | No | No | No | No | No | No |
| Z036 | HTN | II | Non-therapy | No | No | No | No | No | No | No | No | No |
| Z037 | HTN | I | Monotherapy | No | No | No | No | No | Yes | No | No | No |
| Z038 | HTN | II | Non-therapy | No | No | No | No | No | No | No | No | No |
| Z039 | HTN | III | Non-therapy | No | No | No | No | No | No | No | No | No |
| Z040 | HTN | III | Monotherapy | No | Yes | No | No | No | No | No | No | No |
| Z041 | HTN | I | Monotherapy | No | No | Yes | No | No | No | No | No | No |
| Z042 | HTN | I | Bitherapy | No | No | No | No | No | No | No | No | No |
| Z043 | HTN | III | Monotherapy | No | Yes | Yes | No | No | No | No | No | No |
| Z044 | HTN | III | Monotherapy | No | No | Yes | No | No | No | No | No | No |
| Z045 | HTN | III | Monotherapy | No | No | Yes | No | No | No | No | No | No |
| Z046 | HTN | I | Monotherapy | No | Yes | Yes | No | No | No | No | No | No |
| Z047 | HTN | III | Bitherapy | No | No | No | Yes | No | No | No | No | No |
| Z048 | HTN | III | Monotherapy | No | No | Yes | No | No | No | No | No | No |
| Z049 | HTN | III | Tritherapy | No | No | No | No | No | No | No | No | No |
| Z050 | HTN | I | Bitherapy | No | No | No | No | No | No | No | No | No |
| Z051 | HTN | I | Bitherapy | No | No | No | No | No | No | No | No | No |
| Z052 | HTN | III | Bitherapy | No | No | No | No | No | No | No | No | No |
| Z053 | HTN | III | Non-therapy | No | No | No | No | No | No | No | No | No |
| Z054 | HTN | I | Monotherapy | No | No | Yes | No | No | No | No | No | No |
| Z055 | HTN | II | Monotherapy | No | No | Yes | No | No | No | No | No | No |
| Z056 | HTN | III | Monotherapy | No | No | Yes | No | No | No | No | No | No |
| Z057 | HTN | II | Non-therapy | No | No | No | No | No | No | No | No | No |
| Z058 | HTN | I | Monotherapy | No | No | No | No | No | Yes | No | No | No |
| Z059 | HTN | I | Monotherapy | No | No | No | No | No | Yes | No | No | No |
| Z060 | HTN | III | Bitherapy | No | No | No | No | No | No | No | No | No |
| Z061 | HTN | I | Monotherapy | No | No | Yes | No | No | No | No | No | No |
| Z062 | HTN | III | Bitherapy | No | No | No | Yes | No | No | No | No | No |
| Z063 | HTN | II | Monotherapy | No | No | Yes | No | No | No | No | No | No |
| Z064 | HTN | I | Monotherapy | No | No | Yes | No | No | No | No | No | No |
| Z065 | HTN | II | Bitherapy | No | No | No | Yes | No | No | No | No | No |
| Z066 | HTN | I | Monotherapy | No | No | Yes | No | No | No | No | No | No |
| Z067 | HTN | II | Tritherapy | No | No | No | No | No | No | No | No | No |
| Z068 | HTN | I | Non-therapy | No | No | No | No | No | No | No | No | No |
| Z069 | HTN | III | Bitherapy | No | No | No | Yes | No | No | No | No | No |
| Z070 | HTN | I | Monotherapy | No | No | Yes | No | No | No | No | No | No |
| Z071 | HTN | II | Non-therapy | No | No | No | No | No | No | No | No | No |
| Z072 | HTN | III | Non-therapy | No | No | No | No | No | No | No | No | No |
| Z073 | HTN | I | Bitherapy | No | No | No | Yes | No | No | No | No | No |
| Z074 | HTN | II | Monotherapy | No | No | Yes | No | No | No | No | No | No |
| Z075 | HTN | I | Monotherapy | No | No | Yes | No | No | No | No | No | No |
| Z076 | HTN | III | Non-therapy | No | No | No | No | No | No | No | No | No |
| Z077 | HTN | II | Non-therapy | No | No | No | No | No | No | No | No | No |
| Z078 | HTN | III | Monotherapy | No | No | No | No | No | No | No | No | No |
| Z079 | HTN | I | Non-therapy | No | No | No | No | No | No | No | No | No |
| Z080 | HTN | I | Monotherapy | No | No | Yes | No | No | No | No | No | No |
| Z081 | HTN | I | Non-therapy | No | No | No | No | No | No | No | No | No |
| Z082 | HTN | I | Non-therapy | No | No | No | No | No | No | No | No | No |
| Z083 | HTN | I | Monotherapy | No | No | Yes | No | No | No | No | No | No |
| Z084 | HTN | III | Bitherapy | No | No | No | No | No | No | No | No | No |
| Z085 | HTN | II | Monotherapy | No | No | No | No | No | Yes | No | No | No |
| Z086 | HTN | III | Monotherapy | No | No | No | No | No | No | No | Yes | No |
| Z087 | HTN | I | Monotherapy | No | Yes | No | No | No | No | No | No | No |
| Z088 | HTN | I | Monotherapy | No | No | Yes | No | No | No | No | No | No |
| Z089 | HTN | II | Monotherapy | No | No | Yes | No | No | No | No | No | No |
| Z090 | HTN | III | Monotherapy | No | No | Yes | No | No | No | No | No | No |
| Z091 | HTN | II | Bitherapy | No | No | Yes | No | No | No | No | No | No |
| Z092 | HTN | II | Bitherapy | No | No | No | No | No | No | No | No | No |
| Z093 | HTN | III | Monotherapy | No | No | Yes | No | No | No | No | No | No |
| Z094 | HTN | II | Non-therapy | No | No | No | No | No | No | No | No | No |
| Z096 | HTN | I | Non-therapy | No | No | No | No | No | No | No | No | No |
| Te01 | non-HTN | Non-HTN | Non-therapy | No | No | No | No | No | No | No | No | No |
| Te02 | non-HTN | Non-HTN | Non-therapy | No | No | No | No | No | No | No | No | No |
| Te03 | non-HTN | Non-HTN | Non-therapy | No | No | No | No | No | No | No | No | No |
| Te04 | non-HTN | Non-HTN | Non-therapy | No | No | No | No | No | No | No | No | No |
| Te05 | non-HTN | Non-HTN | Non-therapy | No | No | No | No | No | No | No | No | No |
| D150 | non-HTN | Non-HTN | Non-therapy | No | No | No | No | No | No | No | No | No |
| D151 | non-HTN | Non-HTN | Non-therapy | No | No | No | No | No | No | No | No | No |
| D153 | non-HTN | Non-HTN | Non-therapy | No | No | No | No | No | No | No | No | No |
| D154 | non-HTN | Non-HTN | Non-therapy | No | No | No | No | No | No | No | No | No |
| D155 | non-HTN | Non-HTN | Non-therapy | No | No | No | No | No | No | No | No | No |
| D156 | non-HTN | Non-HTN | Non-therapy | No | No | No | No | No | No | No | No | No |
| D157 | non-HTN | Non-HTN | Non-therapy | No | No | No | No | No | No | No | No | No |
| D158 | non-HTN | Non-HTN | Non-therapy | No | No | No | No | No | No | No | No | No |
| D159 | non-HTN | Non-HTN | Non-therapy | No | No | No | No | No | No | No | No | No |
| D160 | non-HTN | Non-HTN | Non-therapy | No | No | No | No | No | No | No | No | No |
| D161 | non-HTN | Non-HTN | Non-therapy | No | No | No | No | No | No | No | No | No |
| D162 | non-HTN | Non-HTN | Non-therapy | No | No | No | No | No | No | No | No | No |
| D163 | non-HTN | Non-HTN | Non-therapy | No | No | No | No | No | No | No | No | No |
| D164 | non-HTN | Non-HTN | Non-therapy | No | No | No | No | No | No | No | No | No |
| D165 | non-HTN | Non-HTN | Non-therapy | No | No | No | No | No | No | No | No | No |
| D166 | non-HTN | Non-HTN | Non-therapy | No | No | No | No | No | No | No | No | No |
| D167 | non-HTN | Non-HTN | Non-therapy | No | No | No | No | No | No | No | No | No |
| D168 | non-HTN | Non-HTN | Non-therapy | No | No | No | No | No | No | No | No | No |
| D169 | non-HTN | Non-HTN | Non-therapy | No | No | No | No | No | No | No | No | No |
| D170 | non-HTN | Non-HTN | Non-therapy | No | No | No | No | No | No | No | No | No |
| D171 | non-HTN | Non-HTN | Non-therapy | No | No | No | No | No | No | No | No | No |
| D172 | non-HTN | Non-HTN | Non-therapy | No | No | No | No | No | No | No | No | No |
| D174 | non-HTN | Non-HTN | Non-therapy | No | No | No | No | No | No | No | No | No |
| D175 | non-HTN | Non-HTN | Non-therapy | No | No | No | No | No | No | No | No | No |
| D176 | non-HTN | Non-HTN | Non-therapy | No | No | No | No | No | No | No | No | No |
| D177 | non-HTN | Non-HTN | Non-therapy | No | No | No | No | No | No | No | No | No |
| D178 | non-HTN | Non-HTN | Non-therapy | No | No | No | No | No | No | No | No | No |
| D179 | non-HTN | Non-HTN | Non-therapy | No | No | No | No | No | No | No | No | No |
| D180 | non-HTN | Non-HTN | Non-therapy | No | No | No | No | No | No | No | No | No |
| D181 | non-HTN | Non-HTN | Non-therapy | No | No | No | No | No | No | No | No | No |
| D182 | non-HTN | Non-HTN | Non-therapy | No | No | No | No | No | No | No | No | No |
| D183 | non-HTN | Non-HTN | Non-therapy | No | No | No | No | No | No | No | No | No |
| D184 | non-HTN | Non-HTN | Non-therapy | No | No | No | No | No | No | No | No | No |
| D185 | non-HTN | Non-HTN | Non-therapy | No | No | No | No | No | No | No | No | No |
| D186 | non-HTN | Non-HTN | Non-therapy | No | No | No | No | No | No | No | No | No |
| D187 | non-HTN | Non-HTN | Non-therapy | No | No | No | No | No | No | No | No | No |
| D188 | non-HTN | Non-HTN | Non-therapy | No | No | No | No | No | No | No | No | No |
| D190 | non-HTN | Non-HTN | Non-therapy | No | No | No | No | No | No | No | No | No |
| D191 | non-HTN | Non-HTN | Non-therapy | No | No | No | No | No | No | No | No | No |
| D193 | non-HTN | Non-HTN | Non-therapy | No | No | No | No | No | No | No | No | No |
| D194 | non-HTN | Non-HTN | Non-therapy | No | No | No | No | No | No | No | No | No |
| D195 | non-HTN | Non-HTN | Non-therapy | No | No | No | No | No | No | No | No | No |
| D196 | non-HTN | Non-HTN | Non-therapy | No | No | No | No | No | No | No | No | No |
| D197 | non-HTN | Non-HTN | Non-therapy | No | No | No | No | No | No | No | No | No |
| D198 | non-HTN | Non-HTN | Non-therapy | No | No | No | No | No | No | No | No | No |
| D199 | non-HTN | Non-HTN | Non-therapy | No | No | No | No | No | No | No | No | No |
| D200 | No | Non-HTN | Non-therapy | No | No | No | No | No | No | No | No | No |

Legend: code with T and Z Hypertensive patients; code with D and Te, Non-hypertensive patients ; HTN : hypertension ; CCB : calcium channel blockers ; ACEI : angiotensin-converting enzyme inhibitor ; ARB2 : angiotensin II receptor blocker. Hypertension was characterized by SBP ≥140 mmHg and PAS ≥80 mmHg. Duration of hypertension varied from 1 to 35 years. Grade 1: Systolic 140 - 159 mm Hg and/or diastolic 90 - 99 mm Hg. Grade 2: Systolic 160 - 179 mm Hg or greater and/or diastolic 100 - 109 mm Hg. Grade 3: Systolic 180 mm Hg or greater and/or diastolic 110 mm Hg or greater. Duration of hypertensive medications varied from 1 to 22 years.

Material : research questionnaire form

Method : we used a questionnaire form to collect dadt on hypertension status, garde, type of therapy, and the différent classes of hypertensive medications used by hypertensive participants.

Suppl Table 3**. Patients, *S. aureus* infection and antibiogram**

| Patient codes | Antibiotics and susceptibility profile | | | | | | | | | | | | | | Resistance profile | *S. aureus* status |
| --- | --- | --- | --- | --- | --- | --- | --- | --- | --- | --- | --- | --- | --- | --- | --- | --- |
|  | AZM | TOB | FOX | FUS | GEN | NIT | CIP | COT | DOX | TET | MIN | CLN | OXA | ERY |  |  |
| T004 | I | S | R | R | S | S | I | R | R | S | R | R | R | R | MDR | MRSA |
| T013 | I | S | R | R | S | S | I | R | R | R | R | R | R | R | MDR | MRSA |
| T016 | R | S | R | R | S | S | I | S | R | R | I | R | R | R | MDR | MRSA |
| T020 | R | S | R | R | S | S | I | R | R | R | R | R | R | R | MDR | MRSA |
| D206 | R | S | S | R | S | S | I | S | R | S | S | R | S | S | MDR | MSSA |
| T036 | S | S | R | S | R | S | S | S | R | R | S | R | S | S | MDR | MSSA |
| T044 | R | R | R | R | S | R | I | R | S | R | R | R | R | R | MDR | MRSA |
| T045 | I | S | R | R | R | R | S | R | R | R | S | R | R | R | MDR | MRSA |
| T060 | R | S | R | R | S | R | R | R | S | R | R | R | R | R | MDR | MRSA |
| T064 | R | S | R | R | S | S | I | R | S | R | S | S | R | R | MDR | MRSA |
| T073 | I | R | R | R | S | S | S | S | R | R | R | R | S | R | MDR | MSSA |
| T076 | I | S | S | R | S | S | S | S | R | R | R | S | R | S | MDR | MRSA |
| T078 | S | S | R | R | S | R | R | R | S | R | R | S | R | R | MDR | MRSA |
| T079 | I | S | S | R | R | R | R | R | R | R | R | R | R | R | MDR | MRSA |
| T080 | R | R | R | R | R | R | R | S | R | R | R | R | R | R | MDR | MRSA |
| T082 | R | R | S | R | R | R | R | R | R | R | R | R | R | R | MDR | MRSA |
| T083 | S | S | S | R | S | S | I | S | R | R | R | R | R | R | MDR | MRSA |
| T084 | I | R | R | R | S | R | I | R | R | R | S | S | R | R | MDR | MRSA |
| T086 | R | R | R | R | R | S | R | S | R | R | R | R | R | R | MDR | MRSA |
| T087 | R | R | S | S | R | R | R | R | R | I | R | R | R | R | MDR | MRSA |
| T091 | R | R | R | R | S | S | R | R | R | R | R | R | R | R | MDR | MRSA |
| T095 | S | R | R | S | R | S | R | S | R | R | R | R | R | I | MDR | MRSA |
| T097 | R | R | S | S | S | R | I | R | R | R | R | R | R | R | MDR | MRSA |
| T104 | R | S | R | R | R | S | R | I | R | S | S | R | R | I | MDR | MRSA |
| T121 | R | R | R | S | S | R | R | R | S | S | R | R | S | R | MDR | MSSA |
| T128 | S | S | R | R | R | R | R | R | S | S | S | S | S | S | MDR | MSSA |
| T132 | R | S | S | R | S | S | R | S | R | I | R | R | R | R | MDR | MRSA |
| T133 | R | S | S | R | S | S | R | S | R | R | R | R | R | R | MDR | MRSA |
| T136 | S | S | S | R | S | S | I | R | R | R | R | R | R | R | MDR | MRSA |
| T138 | S | R | S | R | R | S | I | S | R | S | R | R | R | R | MDR | MRSA |
| T147 | R | R | R | R | R | R | I | R | R | R | R | R | R | R | MDR | MRSA |
| T148 | S | R | R | S | S | R | R | R | R | R | S | S | S | S | MDR | MSSA |
| T153 | R | R | R | S | S | R | I | R | R | R | R | R | R | R | MDR | MRSA |
| T164 | S | R | R | R | S | S | R | R | R | R | R | R | R | R | MDR | MRSA |
| T166 | R | R | S | S | S | S | R | R | R | R | R | R | R | R | MDR | MRSA |
| T170 | S | S | S | R | S | R | I | S | R | I | R | R | R | R | MDR | MRSA |
| T171 | R | R | R | R | S | S | R | R | R | R | R | R | R | R | MDR | MRSA |
| T173 | S | S | R | S | S | S | I | S | R | R | R | R | R | S | MDR | MRSA |
| T174 | R | S | R | S | S | S | I | S | R | R | R | S | R | R | MDR | MRSA |
| T180 | S | S | S | R | S | S | I | S | R | R | R | R | R | R | MDR | MRSA |
| T184 | R | S | S | R | S | R | I | S | R | R | R | R | R | R | MDR | MRSA |
| T212 | S | S | R | R | S | R | R | R | R | R | S | R | R | S | MDR | MRSA |
| T215 | S | S | R | R | S | R | R | R | R | R | R | R | R | R | MDR | MRSA |
| T227 | R | R | R | R | R | S | R | R | R | R | R | R | R | R | MDR | MRSA |
| T229 | R | R | R | R | R | S | I | R | S | S | R | R | S | R | MDR | MSSA |
| D009 | S | R | S | R | R | S | I | I | R | R | S | R | R | R | MDR | MRSA |
| D018 | I | S | R | R | S | S | S | I | R | R | R | R | S | R | MDR | MSSA |
| D020 | R | R | S | S | S | S | S | R | S | R | S | S | R | R | MDR | MRSA |
| D021 | R | R | R | R | R | S | R | I | R | R | R | R | R | R | MDR | MRSA |
| D025 | R | R | R | S | S | R | S | I | R | R | I | R | R | R | MDR | MRSA |
| D031 | R | S | S | R | S | S | S | I | R | I | R | R | S | R | MDR | MSSA |
| D039 | R | S | R | R | S | R | S | I | R | R | R | R | R | R | MDR | MRSA |
| D041 | R | R | R | S | S | S | R | I | R | R | I | R | S | R | MDR | MSSA |
| D047 | R | S | S | S | S | S | R | I | R | R | R | R | R | R | MDR | MRSA |
| D048 | R | R | S | S | S | S | S | R | R | S | R | R | R | R | MDR | MRSA |
| D049 | S | S | S | R | S | S | S | S | S | S | R | R | S | R | MDR | MSSA |
| D050 | S | S | S | S | S | S | S | I | R | R | R | R | S | S | non-MDR | MSSA |
| D051 | I | S | R | S | S | R | S | I | S | R | R | S | S | R | MDR | MSSA |
| D052 | R | S | R | S | S | S | S | I | R | R | R | R | S | R | MDR | MSSA |
| D054 | R | R | R | R | S | S | R | I | R | R | R | R | R | S | MDR | MRSA |
| D055 | R | S | R | R | S | R | R | R | R | R | R | R | R | R | MDR | MRSA |
| D057 | S | R | S | R | S | S | S | I | S | I | S | S | R | R | MDR | MRSA |
| D058 | S | S | S | S | S | S | S | I | S | S | R | R | S | R | non-MDR | MSSA |
| D060 | R | S | S | R | S | R | I | R | R | R | R | S | S | R | MDR | MSSA |
| D074 | R | R | R | S | S | R | S | I | S | R | S | R | S | R | MDR | MSSA |
| D096 | R | S | S | R | S | S | S | R | R | R | R | R | R | R | MDR | MRSA |

Legend: code with T, Hypertensive patients; code with D, non-hypertensive patients **;** GEN: gentamicin ; TOB : tobramycin ; TET: tetracyclin ; CLN : clindamycin ; ERY : erythromycin ; FUS :fusidic acid ; MIN : Minocyclin ; FOX :cefoxitin ; NIT : nitrofurantoin ; COT : cotrimoxazole ; CIP : ciprofloxacin ; OXA : oxacillin ; DOX : doxycyclin ; AZM : azytromycin  S: sensitive; I: intermediate; R: resistant; MRD : Multidrug-resistant ; MSSA: methicillin-sensitive *Staphylococcus aureus*; MRSA: methicillin-resistant *Staphylococcus aureus.* MDR: resistant to at least 3 antibiotics. Non-MDR: resistant to less than 3 antibiotics.

**Materials :** antibiotic disks (cefoxitin, oxacillin, ciprofloxacin, gentamycin, tobramycin, tetracycline, minocycline, doxycycline, clindamycin, erytromycin, azithromycin, fusidic acid, nitrofurantoin, and cotrimoxazole); Mueller-Hinton agar; petri dish; cotton swab; cefoxitin disc.

**Method :** *In vitro* susceptibility testing of *Staphylococcus aureus* isolates to various standard antibiotics was carried out using the Kirby-Bauer diffusion method (A.W. Bauer, 1966). The entire MHA surface was swabbed. The inoculation was allowed to dry for 15 minutes, followed by the deposition of the antibiotic discs. The plates were then incubated at 37 °C for 24 hours. After incubation, the zones of inhibition around the antibiotic discs were measured and interpreted according to Clinical and Laboratory Standards Institute (CLSI) criteria (CLSI, 2023). The susceptibility to methicillin was tested using a 30-µg cefoxitin disc. An inhibition halo below 25 mm was considered methicillin-resistant. Bacterial isolates showing resistance to three or more antibiotic families were multi-resistant bacteria (M. Almakrami *et al.,* 2024, R. Laxminarayan *et al.,* 2013).

**References:**

A.W. Bauer, W.M. Kirby, J.C. Sherris, and M. Turck. "Antibiotic susceptibility testing by a standardized single disk method," *Am J Clin Pathol, vol.* 45, no. 4, pp. 493-496, 1966.

C.a.L.S.I. (CLSI). "Performance standard for antimicrobial susceptibility testing. (2023),"  [*https://iacld.com/UpFiles/Documents/672a1c7c-d4ad-404e-b10e-97c19e21cdce.pdf*](https://clsiorg/media/1469/m100s27_samplepdf)  *vol.* Accessed on Febuary 16, 2024.

M. Almakrami, M. Salmen, Y. A. Aldashel, M. H. Alyami, N. Alquraishah, M. Alzureea, and J. Almakramie. "Prevalence of multidrug-, extensively drug-, and pandrug-resistant bacteria in clinical isolates from King Khaled Hospital, Najran, Saudi Arabia, " *Discover Medicine*, vol. 1, pp. 108, 2024.

R. Laxminarayan, A. Duse, C. Wattal, A. K. M. Zaidi, H. F. L. Wertheim, N. Sumpradit, et al. Antibiotic resistance—the need for global solutions, *The Lancet Infectious Diseases*, vol. 13, no. 12, pp. 1057–1098, 2013.
